# LOEN: Lensless opto-electronic neural network empowered machine vision

**DOI:** 10.1038/s41377-022-00809-5

**Published:** 2022-05-04

**Authors:** Wanxin Shi, Zheng Huang, Honghao Huang, Chengyang Hu, Minghua Chen, Sigang Yang, Hongwei Chen

**Affiliations:** Beijing National Research Center for Information Science and Technology (BNRist), Department of Electronic Engineering, Tsinghua University, Beijing, 100084 China

**Keywords:** Imaging and sensing, Photonic devices

## Abstract

Machine vision faces bottlenecks in computing power consumption and large amounts of data. Although opto-electronic hybrid neural networks can provide assistance, they usually have complex structures and are highly dependent on a coherent light source; therefore, they are not suitable for natural lighting environment applications. In this paper, we propose a novel lensless opto-electronic neural network architecture for machine vision applications. The architecture optimizes a passive optical mask by means of a task-oriented neural network design, performs the optical convolution calculation operation using the lensless architecture, and reduces the device size and amount of calculation required. We demonstrate the performance of handwritten digit classification tasks with a multiple-kernel mask in which accuracies of as much as 97.21% were achieved. Furthermore, we optimize a large-kernel mask to perform optical encryption for privacy-protecting face recognition, thereby obtaining the same recognition accuracy performance as no-encryption methods. Compared with the random MLS pattern, the recognition accuracy is improved by more than 6%.

## Abstract (web summary)

This work highly decreases the size, power, and computation for machine vision with novel joint-optimized opto-electronic neural networks.

## Graphical abstract

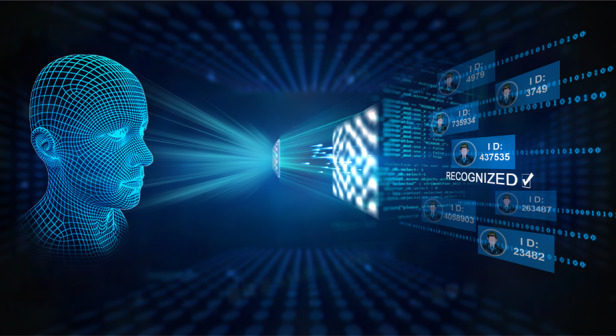

## Introduction

In recent years, owing to the advancements in the immense processing ability and parallelism of modern graphics processing units (GPUs), deep learning^1^ based on convolutional neural networks (CNN) has developed rapidly, leading to effective solutions for a variety of issues in artificial intelligence applications, such as image recognition^2^, object classification^3^, remote sensing^4^, microscopy^5^, natural language processing^6^, holography^7^, autonomous driving^8^, smart homes^9^ and many others^10,11^. However, despite the exponentially increasing computing power, the massive amounts of data involved in vision processing limit the application of CNNs to those portable, power-efficient, computation-efficient hardware to process data on site.

Several studies have been conducted in the field of optical computing to overcome the challenges of electrical neural networks. Optical computing has many appealing advantages, such as optical parallelism, which can greatly improve computing speed, and optical passivity can reduce energy cost and minimize latency. Optical neural networks (ONNs)^12–27^ provide a way to increase computing speed and overcome the bandwidth bottlenecks of electrical units. An ONN can be categorized as a diffraction neural network (DNN)^12–21^, a coherent neural network^22–25^, or a spiking neurosynaptic network^26–29^. Recently, passive ONN schemes for machine vision have been proposed that perform all-optical inference and classification tasks. ONNs have become an alternative to electrical neural networks due to their parallelism and low energy cost. However, previously developed ONNs require a coherent laser as the light source for computation and can hardly be combined with a mature machine vision system in natural light scenes. To further improve the inference capabilities for machine vision tasks, opto-electronic hybrid neural networks^30–34^, in which the front end is optical and the back end is electrical, have been proposed. Lens-based optical architectures mostly complete traditional imaging^34^ or perform some network computing functions^30–33^, such as convolution calculations based on Fourier transform theory. These lens-based systems increase the difficulty of use in edge devices, such as autonomous vehicles. Meanwhile, image capture and image signal processing still account for the majority of the total energy consumption associated with the tasks of opto-electronic hybrid neural network. In fact, all edge devices would benefit from more streamlined systems, with resulting decreases in size, weight, and power consumption^20,35,36^.

**Fig. 1:**
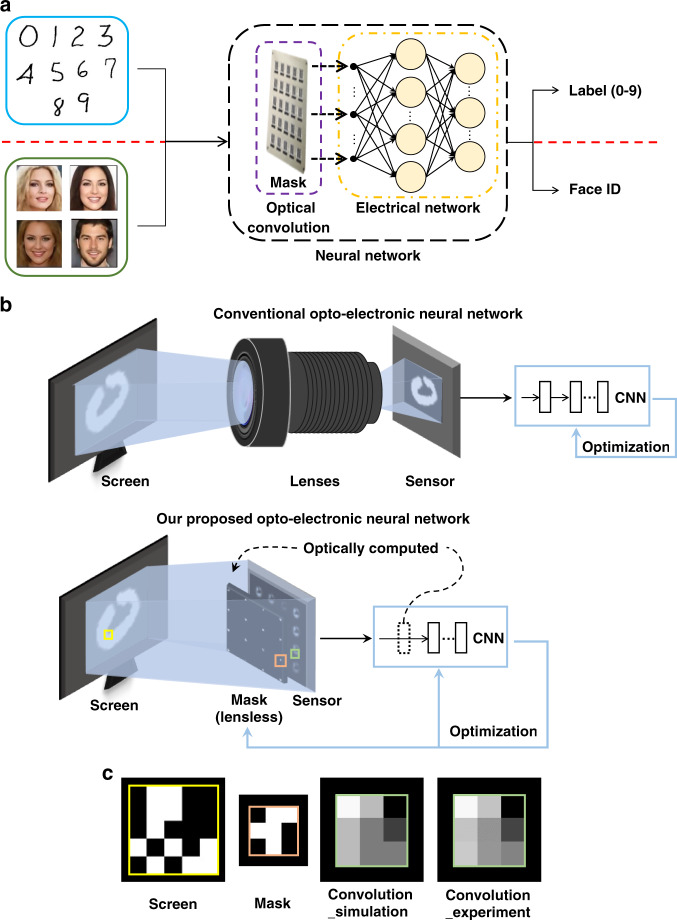
LOEN: Lensless opto-electronic neural network. **a** Through machine learning training and joint optimization, LOEN acquires the ability to complete classification and face recognition tasks. **b** Comparison of the hardware of the conventional and our proposed LOEN. **c** Principle and calibration of convolution process by optical mask

In this paper, we propose a lensless opto-electronic neural network (LOEN) architecture for computer vision tasks that utilizes a passive mask to perform computing in the optical field and addresses the challenge of processing incoherent and broadband light signals in natural scenes (Fig. 1). In addition, the optical link, image signal processing, and back-end network are smoothly combined to achieve joint optimization for specific tasks to reduce calculation effort and energy consumption throughout the entire pipeline. Compared to electrical neural networks or opto-electronic neural networks, our optical link performs established computing functions, such as optical convolution, using only an optical mask and an imaging sensor without a lens. Furthermore, LOEN can operate under incoherent light such as natural light. The structure of the mask is determined by a pre-trained opto-electronic neural network for a specific task, and the optimized convolution layer weights are applied to the mask. A series of machine vision experiments are conducted to demonstrate the performance of LOEN. For tasks such as object classification, a lightweight network for real-time recognition is built. The mask is used for feature extraction, while the single-kernel mask completes the functional verification, and the multiple-kernel mask improves accuracy. For visual tasks such as face recognition, we propose the selection and design methods of the global convolution kernel, which achieve optical encryption without computational consumption. There is no private information, such as recognizable face information, in any links of the end-to-end network, and user privacy can be protected. LOEN does not have a lens structure, so the volume of the system is significantly reduced, and the simple internal design of the optical mask also reduces its production cost. The novel architecture, which cascades all links of the tasks and jointly optimizes them, has numerous potential applications in many actual scenarios, such as autonomous driving, smart homes, and smart security.

## Results

### Optical mask for convolution layer

**Fig. 2:**
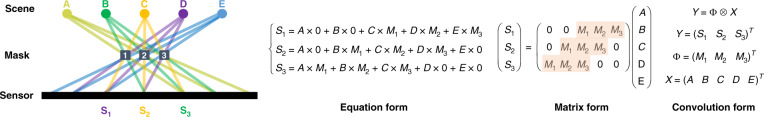
Principle of the optical mask replacing the convolution layer of the network

In this section, we present an optical system that performs the convolution operation of a natural scene with a pre-trained convolution kernel. As shown in Fig. 2, an object can be seen as a surface light source, which can be regarded as a collection of multiple point light sources, such as A, B, C, D, and E. Based on the theory of geometrical optics that light propagates in a straight line, the light from the object transforms through the mask onto the sensor. For instance, the light intensity value *S*_1_ is the sum of the product of the light intensity of the corresponding point on the object and the mask. Transforming from the equation form to the matrix form, the conclusion can be drawn that the light intensity captured by the sensor is the convolution of the object and the mask. In other words, the optical mask can replace the convolution layer of the neural network, and the light intensity distribution captured by the sensor can be regarded as the output of the convolution layer and then given as an input to the remaining layers of the network.

### Network architecture and joint optimization

Figure 1b shows our end-to-end system framework, from the light from real-world scenes (or light on a computer screen) to the recognition result of the network. The framework consists primarily of three components: a mask that implements the first convolution layer of a convolution neural network (CNN), an imaging sensor that captures the output of an optical convolution layer, and a digital processor that completes the following network, which is referred to as the “suffix layers.” Our proposed system removes the first convolution layer of a CNN into the optical domain based on the mask, making the system lensless and greatly reducing the size of the entire system.

**Fig. 3:**
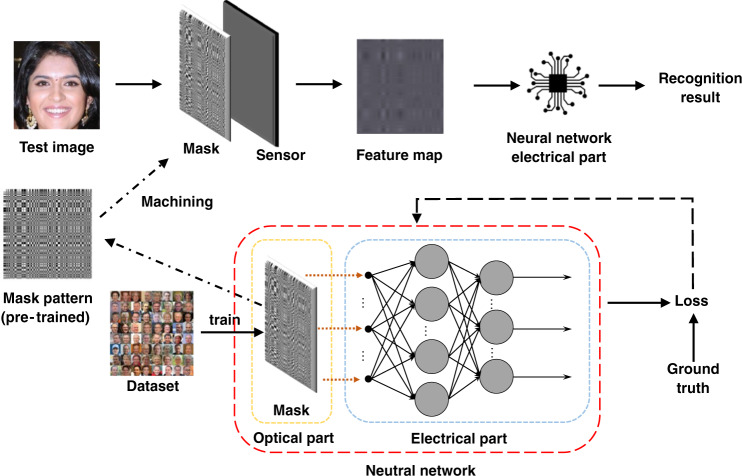
Flow chart of joint optimization

To maximize the overall network recognition accuracy, we jointly optimize the optical convolution layer with the suffix layers in an end-to-end fashion. Figure 3 shows the end-to-end differential pipeline, which incorporates three components: an optical convolution model, a sensor imaging process model, and an electrical network. The convolution kernel and suffix layer weights are the optimization parameters. A loss function (for example, the log loss function) is used to measure the system performance, which is the same as that of an all-electrical neural network.

### Classification and recognition tasks

#### Single-kernel system for MNIST handwritten digit recognition

**Fig. 4:**
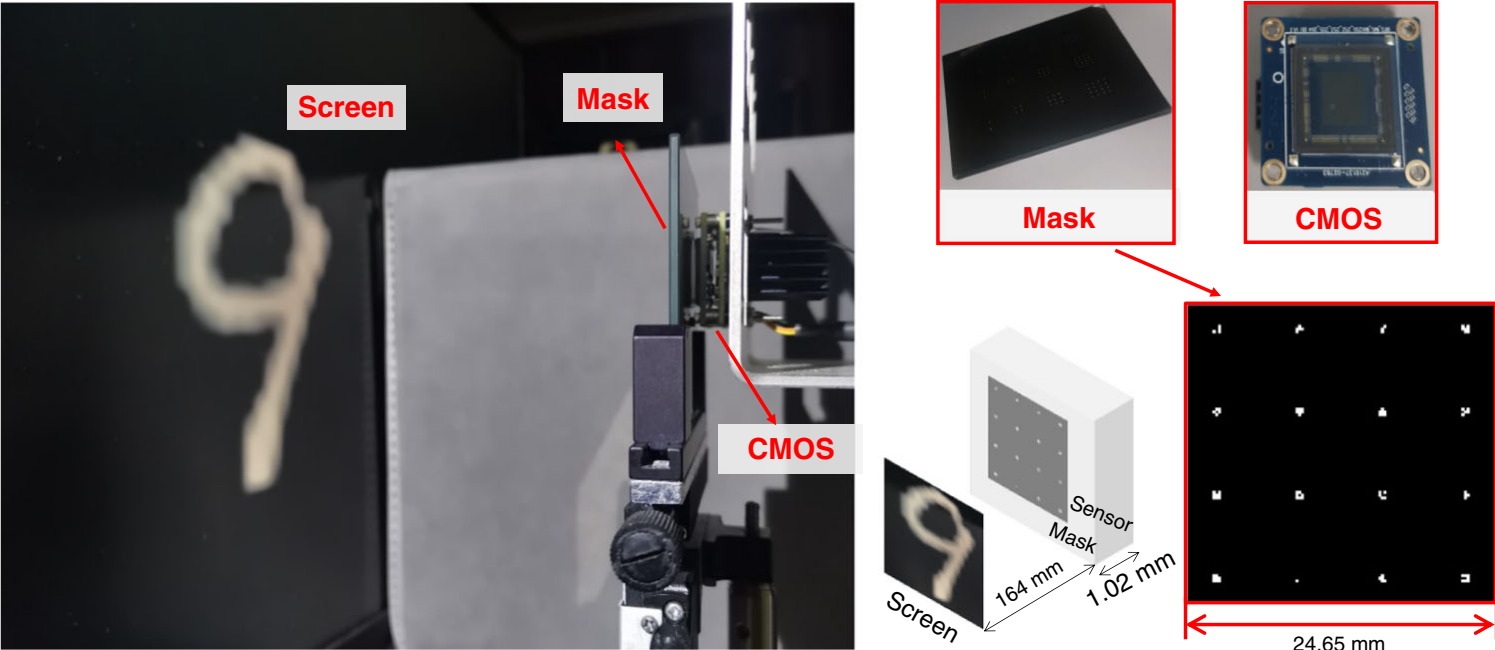
**LOEN prototype for single-kernel and multiple-kernel systems.** Prototype consists of a Sony IMX264 sensor with an optical mask placed approximately 1 mm from the sensor surface

**Fig. 5:**
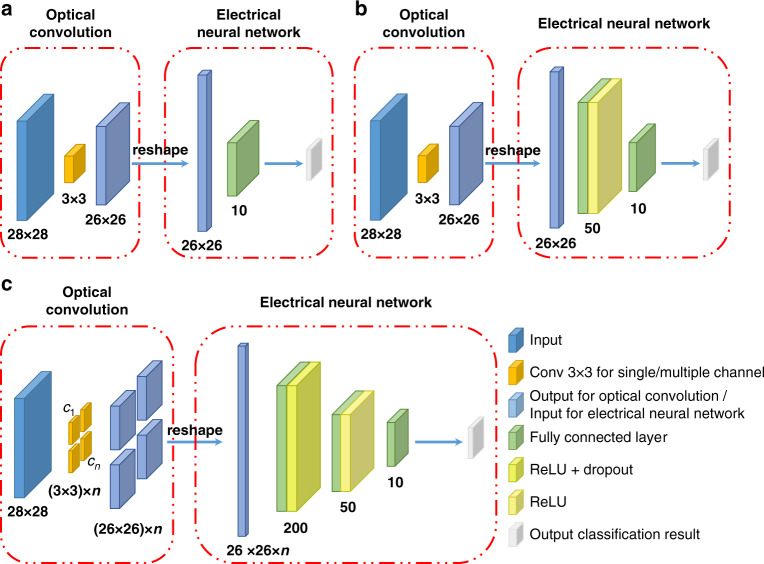
Network architecture for MNIST handwritten digit recognition. **a** Network architecture of a single-kernel convolution neural network based on 1 FC layer. **b** Network architecture of a single-kernel convolution neural network based on 2 FC layers. **c** Network architecture of multiple-kernel convolution neural network. The networks are divided into two parts, and the optical convolution is completed in the optical domain, without calculation and energy consumption

MNIST handwritten digit recognition was selected as the task for the single convolution kernel and multiple convolution kernel systems. For the dataset, we used 60,000 images for training and 10,000 images for testing. As described in the Supplementary Note 5, a feature size of 40 μm was chosen. As shown in the LOEN prototype in Fig. 4, the image was displayed on the computer screen, which was placed 16.4 cm from the optical mask, and the images were pre-compensated for good contrast. As shown in Fig. 5a and b, the entire network consisted of two parts: optical convolution and an electrical neural network. The pixel size of the input image was 28 × 28 and the kernel size of the mask was 3 × 3; hence, the output size of the optical convolution layer was 26 × 26, which was also the input for the electrical network. The electrical network of the architecture of a fully-connected layer (“1 FC layer”) consisted of only 676 input vectors and a linear activation for the output of 10 units, while the electrical network of the architecture of two FC layers (“2 FC layers”) consisted of 676 input vectors, which comprised an FC layer of 50 neurons with a rectified linear unit (ReLu) activation function and the other FC layer with linear activation for the output of 10 units. Because the convolution layer is achieved in the light field, the operations and energy consumption are lower than those of the electrical network with the same architecture.

**Fig. 6:**
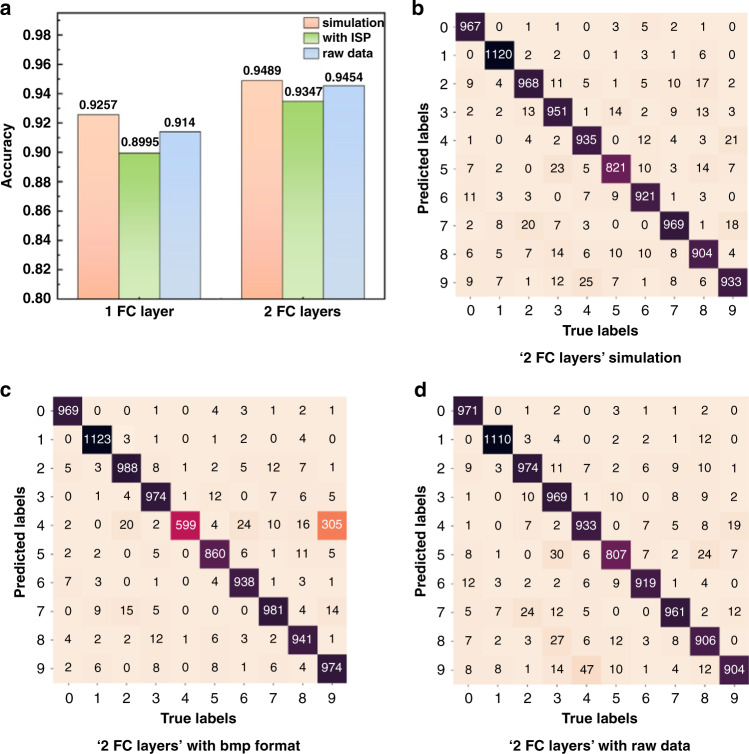
Results for single-kernel system. **a** Experimental recognition accuracy with or without ISP. The feature size was set to 40 μm, and the objects were pre-compensated. **b**–**d** Confusion matrixes for 2 FC layers based on **b** simulation, **c** bmp format data, and **d** raw data, respectively

As shown in Fig. 6a, when a single convolution kernel is used, the recognition accuracy of handwritten digits can reach 89.95% and 93.47%, respectively, under the 1-FC layer and 2-FC layer network structures, respectively. The results are slightly lower than those of the simulator owing to the acquisition noise and convolution calibration deviation (The discussions of the noise model and other parameters effects is shown in the Supplementary Note 8). The computational cost (the number of multiplication addition operations) is reduced by 47.2% compared to the entire electrical network of the 1-FC layer architecture. Meanwhile, the opto-electrical network also saved 47.2% of the energy consumption per image. Similarly, the computational cost and energy consumption were reduced by 15.1% in the 2 FC layers.

We also demonstrated whether image signal processing (ISP) was necessary. The data labeled “raw data” correspond to the accuracy without ISP from the image sensor, while that labeled “with ISP” corresponds to the accuracy with ISP, and the data are captured in bmp format by the sensor. As the results in Fig. 6a show, the accuracy based on raw data was at least approximately 1% higher than that based on the bmp format, which shows that in the LOEN structure, the ISP can be removed from the full link.

#### Multiple-kernel system for MNIST handwritten digit recognition

To increase the classification accuracy of the task, we changed a single kernel to multiple kernels under the condition of spatial reusability. Each convolution kernel was a reversed optimization obtained by training the network. The multiple convolution kernels were placed on the mask in square form, and the distance between every two kernels was determined from the object and kernel pixel size.

In general, the pixel size of the object *n* × *n* is larger than that of the mask *m* × *m*, whereas the pixel size of the full convolution kernel size is (*n* + *m* − 1) × (*n* + *m* − 1). Therefore, the spatial distance between each kernel *d*_M_ should satisfy:

1 $$d_M \ge \left( {n + m - 1} \right) \cdot \Delta$$

Based on the simulation described in the Supplementary Note 5, we chose 40 μm as the feature size Δ, and the numbers of convolution kernels were chosen to be 4, 9, and 16. For example, the pixel size of objects in the MNIST dataset was 28 × 28, while the convolution kernel size was 3 × 3, so the size of the full convolution result was 30 × 30. Therefore, the spatial distance between each kernel in this experimental condition should satisfy *d*_*M*_ ≥ 1.2*mm*.

The convolution network for the multi-kernel system is shown in Fig. 5c. The entire architecture consists of two parts, like that of a single-kernel convolution network. Assume that *n* is the number of channels of the convolution kernel. The input size is 28 × 28, and the kernel size is set to 3 × 3; the output of the optical convolution layer (that is, the input of the electrical network) is 26 × 26 × *n*. The electrical part consists of the input vector with 2 FC layers each with 200 and 50 neurons and the ReLu activation function, and one fully connected layer with linear activation for the output of 10 units.

**Fig. 7:**
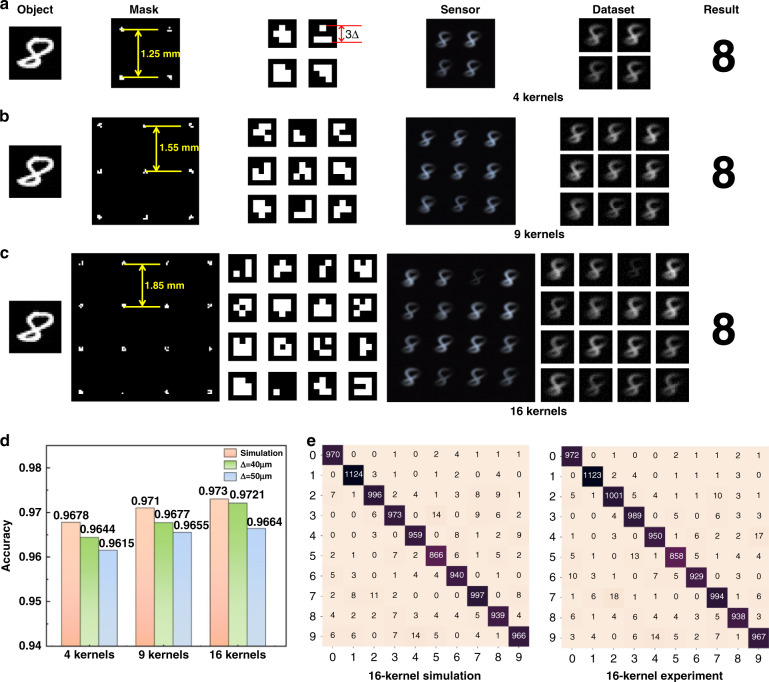
Results for mutiple-kernel systems. Multiple-kernel convolution classification based on **a** 4 kernels, **b** 9 kernels, and **c** 16 kernels. **d** Recognition accuracy for multiple-kernel systems. **e** Confusion matrixes for 16-kernel convolution system

A schematic diagram of each link and the classification accuracy for multiple kernels are shown in Fig. 7. The accuracy for the 16 convolution kernels system can reach 97.21%, which is approximately 3–5% higher than that of the single-kernel system, and the corresponding accuracy of the 40-μm feature size is higher than that of the 50-μm system, which proves the conclusion in Fig. S4e. It should also be noted that the 16-kernel LOEN saved 2.7% of the energy consumption per image, and when the convolution size was larger, the energy consumption was further reduced.

#### Large-kernel system for privacy-protecting face recognition

**Fig. 8:**
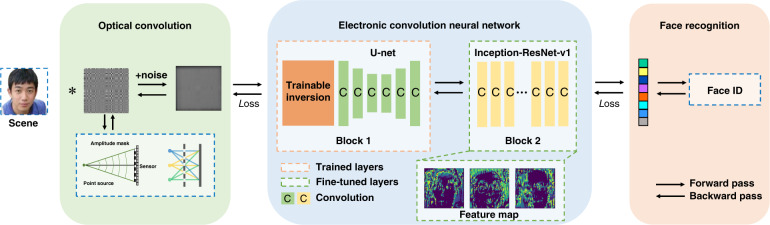
Proposed end-to-end framework for privacy-protecting face recognition. The optical part consists of a sensor and an amplitude mask to achieve encryption by optical convolution. The electrical part consists of two blocks to extract features. We achieve face recognition by jointly optimizing the optics and training the electronic convolution neural network

When using a large-kernel convolution, the lensless system provides a natural condition for privacy protection. Our general strategy is to jointly optimize the lensless optical system and the face recognition network. Specifically, we optimize the mask to degenerate the image and conceal the identity while retaining the features required by the face recognition task as much as possible. To achieve this, we built an end-to-end framework that consists of two parts, as shown in Fig. 8.

In the optical part, we used a designed mask to modulate the incident light amplitude. In the electrical part, we used a deep convolutional neural network to extract features and realize face recognition. The optical and electrical parts were jointly optimized to obtain a pattern suitable for the system and task. The lensless system not only reduces the size and cost of the imaging element but also encrypts the scene using optical convolution. Our lensless privacy-protecting imaging system includes a sensor with a pixel size of 3.45 μm. We place the designed mask close to the sensor; thus, the distance between the mask and sensor is determined by the thickness of the glass on the sensor surface. The pattern of the mask is a square with a length of 510, and the feature size is 10 μm. The feature extraction network consists of a trainable inversion, a U-net backbone (Block1), and an Inception-Resnet-v1 backbone (Block2). For trainable inversion, the initial value of the point spread function (PSF) can be calibrated according to the optical system; otherwise, it is iterated from the pattern directly. In Block 2, we use the model pre-trained on ImageNet as the initial weights to avoid overfitting. During training, we alternately opened Block1 and Block2. Subsequently, the extracted features were input into the classifier to identify the ID.

It is necessary to propose a suitable loss function for jointly optimizing LOEN. The intention is to optimize the PSF to extract more accurate features to achieve an identity classification vision task. Therefore, we used several weighted sums of loss as the total loss function of the training process. The losses used in the model are as follows:

#### Mean squared error (MSE)

We use MSE to measure the gap between the enhanced output of Block1 and the ground truth. Assuming the ground truth image *I*_*groundtruth*_ and the Block1 output *I*_*block*1_, this is given as:

2 $$L_{MSE} = \left\| {I_{groundtruth} - I_{block1}} \right\|_2^2$$

#### Perceptual loss

To evaluate the face recognition feature extraction in Block1, we use perceptual loss to measure the cosine difference (after Block2) between the ground truth and the enhanced output of Block1. Assuming the enhanced feature vector *ϕ*_*block*1_ and the ground-truth image feature vector *ϕ*_*groundtruth*_, this is given as:

3 $$L_{perceptual} = 1 - \cos \left( {\phi _{block1} - \phi _{groundtruth}} \right)$$

#### Negative Log Likelihood (NLL) loss

To evaluate the accuracy of classification, we use NLL loss to measure the gap between the output of the classifier and the target. Assuming the probability distribution P and label *y*_*k*_, this is given as

4 $$L_{NLL} = - \frac{1}{N}\mathop {\sum}\limits_{k = 1}^N {y_k\left( {\log \left( {P_k} \right)} \right)}$$

#### Triplet loss

In face recognition, triple loss minimizes the distance between samples of the same category (an anchor and a positive) and maximizes the distance between samples of different categories (an anchor and a negative). Assuming an anchor denoted as *a*, positive samples denoted as *p*, negative samples denoted as *n*, and the margin as a constant, the triplet loss is given as:

5 $$L_{triplet} = \max \left( {d\left( {a,p} \right) - d\left( {a,n} \right) + {{{\mathrm{margin}}}},0} \right)$$

Finally, the total loss for joint optimization in our framework is given as

6 $$L = \lambda _1L_{MSE} + \lambda _2L_{perceptual} + \lambda _3L_{NLL} + \lambda _4L_{triplet}$$

We first perform an optical simulation by convolving the images of the face with the PSF to obtain an optically encrypted image. Next, Block1 and Block2 extract features from the encrypted image. To simulate the real optical process, we considered the light intensity attenuation due to distance and noise. During training, we used a dataset containing 1000 Face IDs, 50,000 images as the training set, and 5000 images as the test set.

**Fig. 9:**
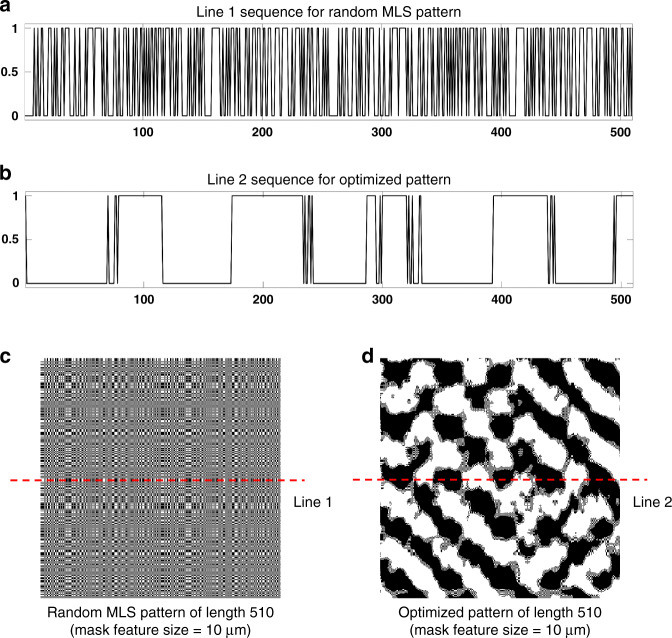
Mask used in our experiment. **a** Line 1 (the 255^th^ line) sequence of random MLS pattern; **b** Line 2 (the 255^th^ line) sequence of our optimized pattern; **c** random MLS pattern of length 510; **d** our optimized pattern of length 510

We optimized a square pattern with a length of 510 and compared the optimized mask pattern with a mask of the same size using a random binary pattern that opened 50% of the features. The random binary pattern is produced by repeating the maximum length sequence (MLS). We set the MLS pattern at a 50% open rate; increasing the number of transparent features beyond 50% deteriorates the conditioning of the system^37,38^. Figure 9 shows the random pattern and the optimized pattern. As shown in the sequence, the mask we optimized maintains a more appropriate luminous flux as the MLS pattern. The peak signal-to-noise ratio (PSNR) and the structural similarity index measure (SSIM) are used to evaluate the gap between the images before and after encryption and the degree of privacy protection^39^. We expect to obtain low PSNR and SSIM while obtaining a high face recognition accuracy.

Table 1 reports the degree of privacy protection and face recognition accuracy in the simulation and experiment. Compared with the MLS pattern, both in simulation and experiment, our optimized pattern leads to better performance in privacy-protecting face recognition, and the recognition accuracy of the optimized pattern is more than 6% better than the MLS pattern. Moreover, the recognition accuracy achieved by our optimized mask is very close to the result without encryption. The gap between simulation and experiment may be due to hardware errors. The increase of the dataset will further improve the ability of fine-tuning in the backend neural network and improve the robustness of the system.

**Table 1:** Privacy protection degree and face recognition accuracy in our method and MLS pattern

Method	Encryption	PSNR	SSIM	Simulation accuracy	Experiment accuracy
Ground truth	✗	—	—	73.6%	—
MLS pattern	✓	10.534	0.107	67.4%	65.1%
Optimized pattern	✓	11.378	0.049	72.8%	71.6%

**Fig. 10:**
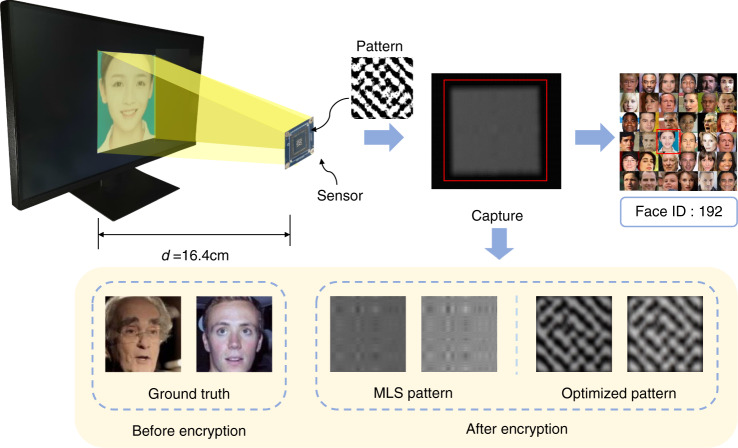
LOEN prototype for large-kernel system to achieve privacy-protecting face recognition

To evaluate the effectiveness of the proposed privacy-protecting face recognition system, we built an optical system. The experimental setup was similar to that shown in Fig. 4. The LOEN prototype for the large kernel system is shown in Fig. 10. Our sensor was a Sony IMX264 placed at a distance of 16.4 cm from the recognized object. After calibrating the system, we obtained the measured PSF as the initial value for Block1. Finally, we obtained the face ID after training Block1 and fine-tuning Block2.

The speed of completing an optical convolution to achieve face encryption is the speed of light. Compared with completing electrical convolution operations based on the same kernel size, the calculation time and the amount of calculation are significantly reduced. In our system, the total time to complete optical encryption and identification is approximately 23 ms. LOEN has the potential to achieve real-time face recognition.

## Discussion

LOEN has been proposed to simplify machine vision tasks without imaging. The entire pipeline consists of optical and electrical parts that are jointly optimized. The convolution is realized in the optical domain using pre-designed masks. Two types of machine vision tasks have been demonstrated for optical convolution and optical encryption. In the MNIST handwritten digit recognition task, the proposed structure used an optical mask to replace the single-kernel or multiple-kernel convolution layer on the electrical field, which achieved 94.54% and 97.21% accuracy, respectively. The computation and energy consumption of the convolution layer were reduced to zero. When considering the entire pipeline, the two components of an imaging pipeline, the sensor and the ISP, have comparable total power costs. The power consumption of imaging sensors ranges from 139 mW for OmniVision OV6922 to 190 mW OmniVision OG02B1B. For ISP, the typical power consumption ranges from 130 mW for the ONsemi AP0101CS image signal processor to 185 mW for the ONsemi AP0100CS image signal processor. Because the two components contribute to the system’s power, the system, when capturing the raw data (without ISP), saves approximately 50% of the energy of traditional pipelines. In the privacy-protecting face recognition task, an optimized optical mask is used to achieve a large-kernel convolution layer and replace digital encryption, which achieves close recognition accuracy performance as the no-encryption methods. Compared with the random MLS pattern, the recognition accuracy is improved more than 6% based on our jointly optimized mask. Meanwhile, there is practically no time cost associated with optical convolution encryption, which enables real-time privacy-protecting face recognition.

LOEN is free of lenses, utilizing parallelism to transform convolution calculations from electrical to optical fields. Unlike DNNs, we are oriented to the visual tasks of the actual scene, not just for optical computation, so the system needs to work with incoherent illumination. All operations for the task are considered jointly. It is expected that the ISP can be optimized for specific functions in more detail to simplify the acquisition process and reduce the power consumption of the sensor. Our approach is based on a single convolution layer. Dynamicity and optical nonlinearity are essential elements of ONNs^19^. When combined with nonlinear materials, such as saturation absorber^40,41^, optical phase change memory^25^ and other novel materials^42^, the nonlinear layer can also be operated on the light field if the material nonlinear threshold can be reached. This enables multiple convolution layers to achieve a closed-loop all-natural-light neural network. The calculation speed is further increased, and the energy consumption is further decreased. When reconfigurable optical elements, such as those based on a liquid crystal modulator^43–45^ or metasurfaces^46,47^, are incorporated into LOEN, the convolution kernels can be programmable. Thus, the convolution in the space and time domains can be realized^48,49^, while the structure can be transferred to other tasks. The method paves the way for a novel solution with small size, intelligence, and low energy consumption to be applied to smart devices for vision tasks.

## Materials and methods

### Optical setup

The system consists of the object (displayed on a screen), a joint-optimized mask, and a CMOS sensor. The sensor used in the experiment is FLIR BFS-U3-51S5C-BD2, and the pixel size is 3.45 μm. The mask is placed close to the sensor. Another critical factor in the system is the feature size. The value of the feature size is determined by many factors, for example, diffraction and geometrical blurs. The specific task and the size of the object will also affect feature size. The detailed discussion is shown in Supplementary Note 1 and Note 5. In addition, the calibration is needed by adjusting the size of the object and the distance between the object and the mask. The calibration of the optical convolution is discussed in Supplementary Note 3.

### Mask selection and fabrication

The mask is obtained by photolithography on a chrome-coated glass substrate. The fabrication process includes photolithography, development, etching, demolding and other steps. The pattern of the mask fabricated in this way is fixed. Another way to form an optical mask is to use a spatial light modulator (SLM), which makes it convenient to adjust the parameters. The selection of the mask type should take the contrast requirement of the machine vision task into consideration. The specific calculations are listed in Supplementary Note 2.

### Dataset processing and neural network training

All the images of the classification and recognition tasks are converted into greyscale and resized to match our system. The networks are trained and tested on a workstation with a 3.3-GHz Intel Core i9-9940X central processing unit (CPU) (32 GB RAM) and two Nvidia GeForce RTX2080Ti GPUs while using the Pytorch framework. The structure and parameters of the Face Recognition task are shown in Supplementary Note 11.

## Supplementary information

Supplementary Information for LOEN: Lensless opto-electronic neural network empowered machine vision

## Data Availability

The custom code and mathematical algorithm used to obtain the results presented in this paper are available from the corresponding author upon reasonable request.

## References

[1] LeCun Y, Bengio Y, Hinton G (2015). Deep learning. Nature.

[2] Krizhevsky, A., Sutskever, I. & Hinton, G. E. Imagenet classification with deep convolutional neural networks. In *Proceedings of the 25th International Conference on Neural Information Processing System*. 1097–1105 (Lake Tahoe, Nevada: Curran Associates Inc, 2012).

[3] He, K. M. et al. Deep residual learning for image recognition. In *Proceedings of 2016 IEEE Conference on Computer Vision and Pattern Recognition*, 770–778 (Las Vegas, NV, USA: IEEE, 2016).

[4] Zhu XX (2017). Deep learning in remote sensing: a comprehensive review and list of resources. IEEE Geosci. Remote Sens. Mag..

[5] Rivenson Y (2017). Deep learning microscopy. Optica.

[6] Young T, Hazarika D, Poria S, Cambria E (2018). Recent trends in deep learning based natural language processing. ieee Comput. Intell. Mag..

[7] Rivenson Y, Wu YC, Ozcan A (2019). Deep learning in holography and coherent imaging. Light Sci. Appl..

[8] Al-Qizwini, M. et al. Deep learning algorithm for autonomous driving using GoogLeNet. In *Proceedings of 2017 IEEE Intelligent Vehicles Symposium (IV)*, 89–96 (Los Angeles, CA, USA: IEEE, 2017).

[9] Shi QF (2020). Deep learning enabled smart mats as a scalable floor monitoring system. Nat. Commun..

[10] Xiong HY (2015). The human splicing code reveals new insights into the genetic determinants of disease. Science.

[11] Helmstaedter M (2013). Connectomic reconstruction of the inner plexiform layer in the mouse retina. Nature.

[12] Li JX (2019). Class-specific differential detection in diffractive optical neural networks improves inference accuracy. Adv. Photonics.

[13] Luo Y (2019). Design of task-specific optical systems using broadband diffractive neural networks. Light Sci. Appl..

[14] Mengu D (2020). Misalignment resilient diffractive optical networks. Nanophotonics.

[15] Kulce O (2021). All-optical information-processing capacity of diffractive surfaces. Light Sci. Appl..

[16] Rahman MSS (2021). Ensemble learning of diffractive optical networks. Light Sci. Appl..

[17] Lin X (2018). All-optical machine learning using diffractive deep neural networks. Science.

[18] Li JX (2021). Spectrally encoded single-pixel machine vision using diffractive networks. Sci. Adv..

[19] Goi E (2021). Nanoprinted high-neuron-density optical linear perceptrons performing near-infrared inference on a CMOS chip. Light Sci. Appl..

[20] Wetzstein G (2020). Inference in artificial intelligence with deep optics and photonics. Nature.

[21] Yan T (2019). Fourier-space diffractive deep neural network. Phys. Rev. Lett..

[22] Shen YC (2017). Deep learning with coherent nanophotonic circuits. Nat. Photonics.

[23] Harris NC (2018). Linear programmable nanophotonic processors. Optica.

[24] Zhang QM (2019). Artificial neural networks enabled by nanophotonics. Light Sci. Appl..

[25] Harris NC (2017). Quantum transport simulations in a programmable nanophotonic processor. Nat. Photonics.

[26] Feldmann J (2019). All-optical spiking neurosynaptic networks with self-learning capabilities. Nature.

[27] Xu XY (2021). 11 TOPS photonic convolutional accelerator for optical neural networks. Nature.

[28] Peng HT (2018). Neuromorphic photonic integrated circuits. IEEE J. Sel. Top. Quantum Electron..

[29] Xu XY (2020). Photonic perceptron based on a Kerr Microcomb for high‐speed, scalable, optical neural networks. Laser Photonics Rev..

[30] Chang JL (2018). Hybrid optical-electronic convolutional neural networks with optimized diffractive optics for image classification. Sci. Rep..

[31] Zhou TK (2021). Large-scale neuromorphic optoelectronic computing with a reconfigurable diffractive processing unit. Nat. Photonics.

[32] Chen, H. G. et al. ASP Vision: optically computing the first layer of convolutional neural networks using angle sensitive pixels. In *Proceedings of 2016 IEEE Conference on Computer Vision and Pattern Recognition*, 903–912 (Las Vegas, NV, USA: IEEE, 2016).

[33] Miscuglio M (2020). Massively parallel amplitude-only Fourier neural network. Optica.

[34] Pad, P. et al. Efficient neural vision systems based on convolutional image acquisition. In *Proceedings of 2020 IEEE/CVF Conference on Computer Vision and Pattern Recognition*, 12282–12291 (Seattle, WA, USA: IEEE, 2020).

[35] LiKamWa, R. et al. Energy characterization and optimization of image sensing toward continuous mobile vision. In *Proceeding of the 11th Annual International Conference on Mobile Systems, Applications, and Services*, 69–82 (Taipei, China: ACM, 2013).

[36] LiKamWa R (2016). RedEye: analog ConvNet image sensor architecture for continuous mobile vision. ACM SIGARCH Computer Architecture N..

[37] Asif MS (2017). FlatCam: Thin, lensless cameras using coded aperture and computation. IEEE Trans. Comput. Imaging.

[38] Khan SS (2020). FlatNet: towards photorealistic scene reconstruction from lensless measurements. IEEE Trans. Pattern Anal. Mach. Intell..

[39] Hinojosa, C., Niebles, J. C. & Arguello, H. Learning privacy-preserving optics for human pose estimation. In *Proceedings of 2021 IEEE/CVF International Conference on Computer Vision*, 2553–2562 (Montreal, QC, Canada: IEEE, 2021).

[40] Dejonckheere A (2014). All-optical reservoir computer based on saturation of absorption. Opt. Express.

[41] Lim GK (2011). Giant broadband nonlinear optical absorption response in dispersed graphene single sheets. Nat. Photonics.

[42] Miscuglio M (2018). All-optical nonlinear activation function for photonic neural networks [Invited]. Optical Mater. Express.

[43] Cheng KT (2016). Electrically switchable and permanently stable light scattering modes by dynamic fingerprint chiral textures. ACS Appl. Mater. Interfaces.

[44] Ke YJ (2019). Smart windows: electro-, thermo-, mechano-, photochromics, and beyond. Adv Energy Mater.

[45] Van der Asdonk P, Kouwer PHJ (2017). Liquid crystal templating as an approach to spatially and temporally organise soft matter. Chem. Soc. Rev..

[46] Li ZL (2017). Dielectric meta-holograms enabled with dual magnetic resonances in visible light. ACS Nano.

[47] Kim I (2018). Outfitting next generation displays with optical metasurfaces. ACS Photonics.

[48] Hu CY (2021). Video object detection from one single image through opto-electronic neural network. APL Photonics.

[49] Hu CY (2021). FourierCam: a camera for video spectrum acquisition in a single shot. Photonics Res..

