# Supplementary material for: LOEN: Lensless opto-electronic neural network empowered machine vision
Source: Light Sci Appl. 2022 May 4;11:121. doi: 10.1038/s41377-022-00809-5 (PMC9068799; doi:10.1038/s41377-022-00809-5)
Supplement: Supplementary file 1 — Supplementary Information for LOEN: Lensless opto-electronic neural network empowered machine vision [file 41377_2022_809_MOESM1_ESM.docx]

Supplementary Information for

LOEN: Lensless opto-electronic neural network empowered machine vision

**Wanxin Shi^#^, Zheng Huang^#^, Honghao Huang, Chengyang Hu, Minghua Chen, Sigang Yang, Hongwei Chen***

* Corresponding author: [chenhw@tsinghua.edu.cn](mailto:chenhw@tsinghua.edu.cn)

# These authors contribute equally to this work.

Affiliation: Beijing National Research Center for Information Science and Technology (BNRist)

Department of Electronic Engineering, Tsinghua University, Beijing 100084, China

**Supplementary Note 1: Optical parameter determination of optical mask**

The optical convolution layer places an amplitude mask immediately before the sensor. We assume that the mask and sensor are planar, parallel to each other, and separated by distance $d_{MS}$. For simplicity of explanation, we assume that the mask modulates light in a binary fashion, while the optical mask consists of transparent parts that transmit light and opaque parts that block light, so the equivalent kernel size is binary, i.e., 0 or 1. We denote the size of transparent/opaque parts features by $\Delta$, which is called the feature size.

As shown in Fig. S1(a), we assume that the distance between two adjacent point lights is $d_{L}$, the distance between the mask and light is $d_{LM}$, and the distance between the mask and sensor is $d_{MS}$. Here, we discuss how to determine the values of the three interrelated parameters: $\Delta$, $d_{LM}$, and $d_{L}$.


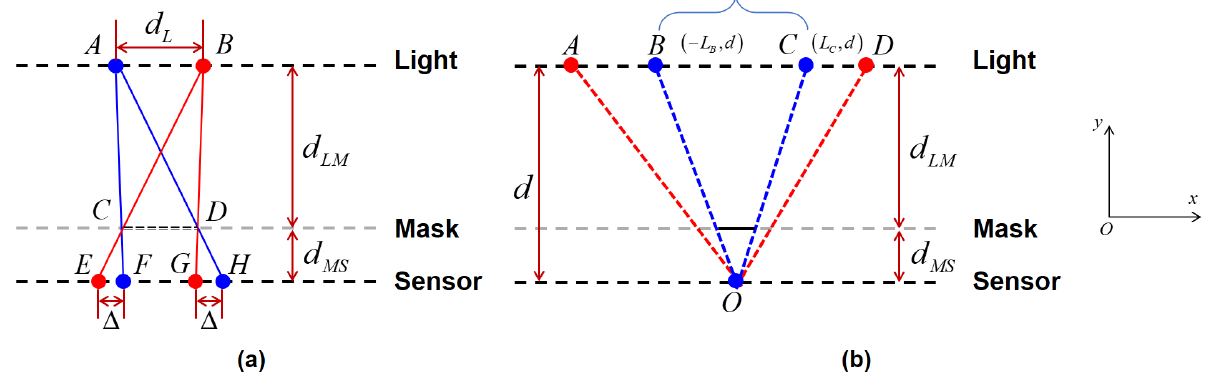


**Fig. S1 Requirements for optical convolution calculation. (a) Determination of the distance between point light sources. (b) Mask contrast requirement for optical convolution calculation.**

Considering light from a light source passing through one of the mask openings, the light distribution recorded at the sensor is the point spread function (PSF). The size of the PSF depends on diffraction and geometrical blurs, which in turn depends on the distance between the mask and the sensor $d_{MS}$ and the feature size of the mask $\Delta$. The size of the diffraction blur is approximately $2.44\lambda d_{MS}/\Delta$, where λ is the wavelength of the light source. The size of the geometrical blue is equal to the feature size $\Delta$. Therefore, the relational expression satisfied by the feature size is as follows:

$\Delta\geq\sqrt{2.44\lambda d}$ (S1)

When the formula takes the equal sign, the total blur of the system is the smallest; however, in practice, due to the greater influence of diffraction, the feature size can be rounded up appropriately in order to simplify the production.

To reduce the size of the PSF, the feature size $\Delta$ should be increased. However, the convolution results take up more space as $\Delta$ increases, while multiplexing within the scene pixels also decreases. In actual experiments, the feature size should be determined while taking the extent of space multiplexing and the distance $d_{MS}$ into consideration.

**Supplementary Note 2: Discussion of the contrast requirement for the optical mask**

The ideal optical mask is a 0–1 binary convolution kernel; however, the true mask cannot completely block the contrasting light. We assume that the contrast of the mask is the ratio of the white and black gray values captured by the sensor. As shown in Fig. S1(b), the light intensity consists of effective and ineffective light intensity. For example, for a point $O$ on the sensor, the light propagates through the mask to point $O$ between $BC$, the effective light intensity, and the light from other points on the light source plane is ineffective light intensity.

The light field intensity propagation formula of a point light source is as follows:

$E\text{=}\frac{A_{0}}{r}\cos\left(\omega t-\vec{k}\vec{r}\right)$ (S4)

where $A_{0}$ is the initial light field intensity constant, $r$ is the distance between the point light source and the measurement point, $\omega$ is the light frequency, and $\vec{k}$ is the wave vector. While the light intensity is inversely proportional to the square of the distance,

$I\propto\frac{1}{r^{2}}$ (S5)

Taking Fig. S1 (b) as an example, the light intensity of $O$ that is generated by point light source $B$ is as follows:

$I_{OB}\text{=}\frac{I_{0}}{d^{2}+L_{B}^{2}}$ (S6)

where $I_{0}$ is the light intensity constant of the point light source. We assume that the contrast of the optical mask is $N$. The light intensity $O$generated by the point light source $A$ is as follows:

$I_{OA}=\frac{1}{N}\cdot\frac{I_{0}}{d^{2}+L_{A}^{2}}$ (S7)

The effective light intensity of point $O$ generated by $BC$ is as follows:

$I_{eff}=\int_{-L_{B}}^{L_{C}} \frac{I_{0}}{d^{2}+L^{2}}dL=\frac{I_{0}}{d}\cdot\arctan\left(\frac{L}{d}\right)\left|_{-L_{B}}^{L_{C}}\right.=\frac{I_{0}}{d}\left[\arctan\left(\frac{L_{C}}{d}\right)+\arctan\left(\frac{L_{B}}{d}\right)\right]$ (S8)

The ineffective light intensity of the point $O$ generated by the other locations of the light source field is as follows:

$I_{ineff}=\int_{-\infty}^{-L_{B}} \frac{1}{N}\cdot\frac{I_{0}}{d^{2}+L^{2}}dL+\int_{L_{C}}^{+\infty} \frac{1}{N}\cdot\frac{I_{0}}{d^{2}+L^{2}}dL=\frac{I_{0}}{Nd}\left[\arctan\left(\frac{L}{d}\right)\left|_{-\infty}^{-L_{B}}+\arctan\left(\frac{L}{d}\right)\left|_{L_{C}}^{+\infty}\right.\right.\right]$ (S9)

According to the triangle similarity theory, the effective range $BC$ of the light source field can be expressed as follows:

$BC\text{=}\frac{d}{d_{MS}}\cdot m\Delta$ (S10)

To simplify the calculation, we assume that the side length of the mask is $m\Delta$ and is fully transparent. We assume that points $B$ and points $C$ are symmetric about the y-axis, which simplifies the calculation of the integration. The equivalent pixel of the light source is $n\times n$, whereas in space, the side length of the light source is $n\times dL$, according to Eq. (S3), and the light intensity at other locations on the light source plane was 0. The effective light intensity of point $O$ is as follows:

$I_{eff}=\frac{2I_{0}}{d}\cdot\arctan\left(\frac{m\Delta}{2d_{MS}}\right)$ (S11)

The expression of the ineffective light intensity of point $O$ is as follows:

$I_{ineff}=\frac{I_{0}}{Nd}\left[\arctan\left(\frac{L}{d}\right)\left|_{-\frac{n}{2}d_{L}}^{-\frac{BC}{2}}+\arctan\left(\frac{L}{d}\right)\left|_{\frac{BC}{2}}^{\frac{n}{2}d_{L}}\right.\right.\right]=\frac{2I_{0}}{Nd}\left[\arctan\left(\frac{L}{d}\right)\left|_{\frac{BC}{2}}^{\frac{n}{2}d_{L}}\right.\right]=\frac{2I_{0}}{Nd}\left[\arctan\left(\frac{n\Delta d_{LM}}{2d_{MS}d}\right)-\arctan\left(\frac{m\Delta}{2d_{MS}}\right)\right]$ (S12)

Because $\frac{d_{LM}}{d_{MS}}>>100\text{ }$, the distance between the mask and the sensor $d_{MS}$ can be ignored; that is, $d_{LM}\approx d$. Thus, Eq. (S12) can be simplified to the following:

$I_{ineff}=\frac{2I_{0}}{Nd}\left[\arctan\left(\frac{n\Delta}{2d_{MS}}\right)-\arctan\left(\frac{m\Delta}{2d_{MS}}\right)\right]$ (S13)

To satisfy the requirement that the convolution result can be detected and recognized, the signal-to-noise ratio should be greater than 1. The experimental parameter values are chosen; for example, the distance between the mask and the sensor $d_{MS}=1.02\text{ }mm$, the equivalent pixel of the light source is $28\times28$; that is,$n=28$; the equivalent pixel of the light source is $3\times3$; that is, $m=3$. The feature size $\Delta$ is set to 40 μm, according to Eq. (S1). The signal-to-noise ratio should satisfy the following:

$I_{eff}>I_{ineff}\Rightarrow N>6.89$ (S14)

For $d_{MS}=1.02\text{ }mm$, $n=28$, and $m=5$, for example, the signal-to-noise ratio should be $N>3.74$; for $d_{MS}=1.02mm$, $n=32$, $m=3$, the signal-to-noise ratio should be $N>8.54$.

Notably, we assume that the light field is ideal and that there is no noise in the actual environment and the capture process of the sensor; therefore, the calculation of the contrast of the mask is ideal. For actual experimental conditions, the contrast of the mask $N$ should be larger to ensure task recognition accuracy.

**Supplementary Note 3: Calibration of the optical convolution layer**

After the determination of $d_{MS}$ and feature size $\Delta$, the two distances $d_{LM}$ and $d_{L}$ can be determined to meet the convolution requirement. According to the convolution principle, when the point light translates by distance $d_{L}$, the pattern captured by the sensor translates by distance $\Delta$. Under this condition, when the light is a surface light in which the distance between two adjacent point light sources is $d_{L}$, the light intensity distribution captured by the sensor is the convolution of the light and mask.

According to the triangle similarity principle, $\Delta ABC\text{\textasciitilde}\Delta FEC$, and the following expression can be obtained:

$\frac{AB}{FE}=\frac{d_{L}}{\Delta}=\frac{d_{LM}}{d_{MS}}$ (S2)

Simplifying the formula:

$d_{L}=\frac{\Delta}{d_{MS}}\cdot d_{LM}$ (S3)

Owing to the determination of $\Delta$ and $d_{MS}$, the distance $d_{L}$ and $d_{LM}$ can be set while satisfying Eq. (S3).


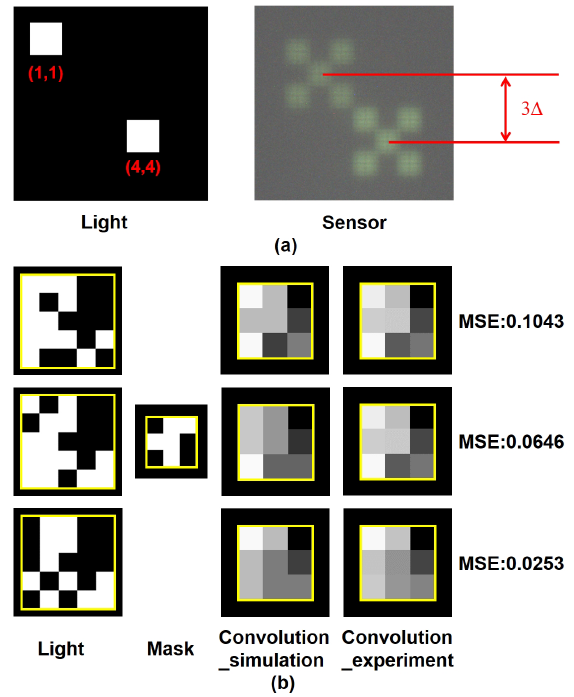


**Fig. S2 (a) Schematic diagram of the convolution calculation correction in the experiment. (b) Result of optical lensless convolution. The “Light” is the picture displayed on the screen, the mask pattern is unchanged in the experiment, and the MSE is the mean square error between the convolution simulation results and the experiment results.**

In the actual experiment, we need to fine-tune the theoretical calculation results to correct the convolution results. As shown in Fig. S2(a), we choose the $3\times3$ cross-type as the optical convolution kernel, and the equivalent light source pixel is $5\times5$. When the gray values of the coordinate positions (1,1) and (4,4) are set to 255, the ideal convolution result will be two cross graphics in which the horizontal and vertical distances between them are both $3\Delta$. After the determination of system parameters such as $d_{MS}$, $\Delta$, $d_{LM}$, and $d_{L}$, the size of the light source is first set to 5$d_{L}$. Then, the size of the $5\times5$ light source is fine-tuned until the distance between the two cross graphics is $3\Delta$ on the sensor, as shown in Fig. S2 (a), and the correction of the optical convolution calculation is performed.

Once the mask and the network are trained, the mask is produced as an optical element, and the rest of the network is set as an electrical processing unit. There are several ways to produce an optical mask fall into two categories: one involves forming a fixed mask structure on the substrate by photolithography technology, and the other involves the use of a spatial light modulator (SLM). We use the SLM in the process of convolution calibration because its electrical programmability makes it convenient to adjust and confirm the parameters. However, the pixel size for the SLM (Holoeye LC 2012 Spatial Light Modulator) used was 36 mm, but the required distance between the mask and the sensor $d_{MS}$, given by Eq. (S1), could not be achieved as it was limited by the SLM structure. The contrast for the SLM is lower than 5, which makes it impossible to capture the signal in a general computer vision task, according to Eq. (S8)–(S14). Therefore, after the convolution calibration and determination of the parameters, the fixed mask on the substrate is used in the subsequent systems facing specific CV tasks. Except for the high contrast (higher than 200), the cost of the fixed mask is lower, making the whole system more compact.

As shown in Fig. S2(b), we choose an $3\times3$ optical mask and three $5\times5$ light sources to perform the convolution calculation. Both the light sources and convolution kernel are assumed to be 0–1 binary. The light sources were displayed on the computer screen. The total calculation process is free of the lens, and the convolution calculation result is directly captured by the imaging sensor. Because the spatial size of a pixel is not equal to the feature size, the average grayscale value of multiple pixels must be taken to obtain the convolution result of the equivalent single pixel. After the simple process described above, the valid convolution result of the light source and the optical mask is obtained. We use the mean squared error (MSE) to quantitatively measure the accuracy of the optical convolution. As shown in the result, the MSE of convolution is less than 0.11, proving that the method can replace the convolution calculation. Owing to the low contrast of the transmissive spatial light modulator, the result will be more accurate if an optical mask is used.

**Supplementary Note 4:** **The spatial resolution of the system**

In note 3, the minimum distance between the adjacent point light source $d_{L}$has already been defined. When the hardware system is fixed, the image spatial resolution has also been determined. The spatial resolution is related to the distance between two light source and the system field of view (FOV). The FOV of the system is limited by the FOV of the imaging sensor. As shown in Fig.S3(a), the FOV of the sensor is assumed as $\theta$. So the field size of the system is:

$L\text{=}d\cdot\tan\frac{\theta}{2}$ (S15)

The image spatial resolution is the ratio of the field size and the distance between two light sources. As discussed above, the distance between the scene and the mask if above 100 mm, while the distance between the mask and the sensor is about 1 mm. Due to the relationship $\frac{d_{LM}}{d_{MS}}>>100\text{ }$, that is $d_{LM}\approx d$, so the expression of the spatial resolution is:

$SR=\frac{L}{dL}=\frac{2d\cdot\tan\frac{\theta}{2}}{\frac{\Delta}{d_{MS}}\cdot d_{LM}}\approx\frac{2d_{MS}\cdot\tan\frac{\theta}{2}}{\Delta}$ (S16)

Take the sensor and parameters in our experiments as an example, an angular response of the sensor can be chosen as 60 degrees [37], which is referred to as the field of the view of the sensor. In the single and multiply kernel experiments, the feature size is 40 μm, so the image spatial resolution is $29.4\times29.4$; while for the large kernel experiment, the feature size is 10 μm, and the image spatial resolution is $117.8\times117.8$. In the actual experiment, the dataset used in single and multiply kernel system is MNIST handwritten digits, and the pixel size of the images is $28\times28$; while in the large kernel system, the human face images are set as $112\times112$. Due to the discussion, the images used for different tasks can be recognized by the system at the same spatial resolution of themselves.


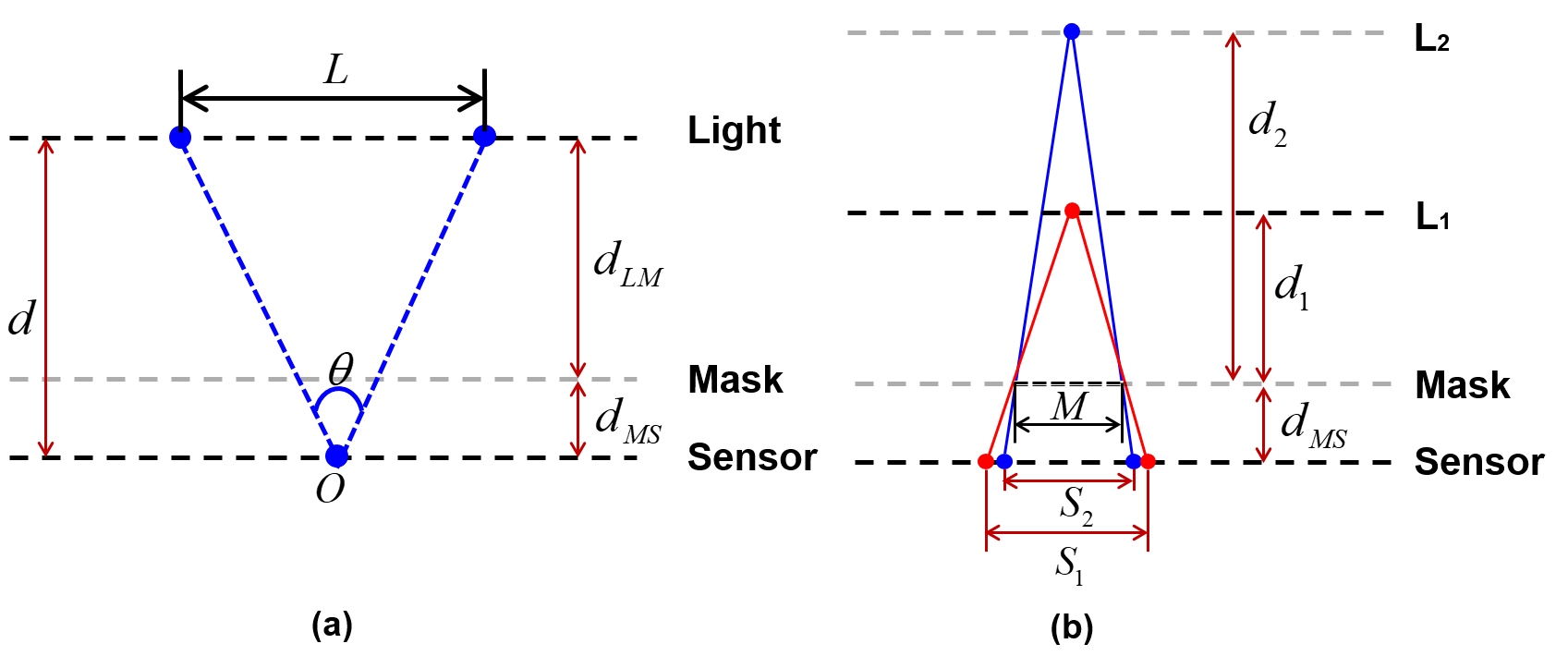


**Fig. S3 (a) Schematic diagram of the field size of the system. (b) Schematic diagram of PSF variation with depth.**

**Supplementary Note 5: Parameter determination in the single-kernel and multiply-kernel system for MNIST handwritten digit recognition task**

In this section, we discuss how different physical parameters affect the performance of the entire network. Optical processing and image post-processing have several effects on network performance. Parameters such as the distance between the light source plane and mask, $d_{LM}$, and the distance between the mask and sensor, $d_{MS}$, are fixed.

Fig. S4 shows the accuracy curves affected by various factors, such as diffraction, noise, and luminous flux. The diffraction effect is determined by the opening area size of the mask; hence, the feature size is chosen as the independent variable when discussing the diffraction factor. Regarding the convolution kernel for the handwritten digit recognition task, according to Fig. S4 (a) and (b), the convolution size should be set to 3, and the performance of the optimized binary kernel is better than that of all individual kernels and random floating-point kernels. Therefore, we choose three optimized binary kernels for the task, and the kernels used in the following discussion and experiment are all $3\times3$ binary, with 0 or 1 values.

The diffraction gradually decreases as the feature size increases, and the convolution kernel is more similar to that on the mask. As shown in Eq. (S1), the threshold for the feature size is approximately 37 μm. As shown in Fig. S4(c), when the feature size is lower than the threshold, the recognition accuracy gradually increases with increasing feature size. However, when the feature size is higher than the threshold, the recognition accuracy tends to stabilize and no longer increases (a slight fluctuation in recognition accuracy is a normal phenomenon of the network).

We selected Gaussian noise to simulate the effect of noise; we set the noise mean value to 0, and σ denotes the standard deviation of the noise. Fig. S4(d) shows the variation curve of the recognition accuracy affected by the Gaussian noise standard deviation. As the noise standard deviation increased, the accuracy tended to gradually decrease (although fluctuations of such networks are normal). Therefore, in the actual experiment, the noise should be controlled as much as possible to ensure the contrast of the system.


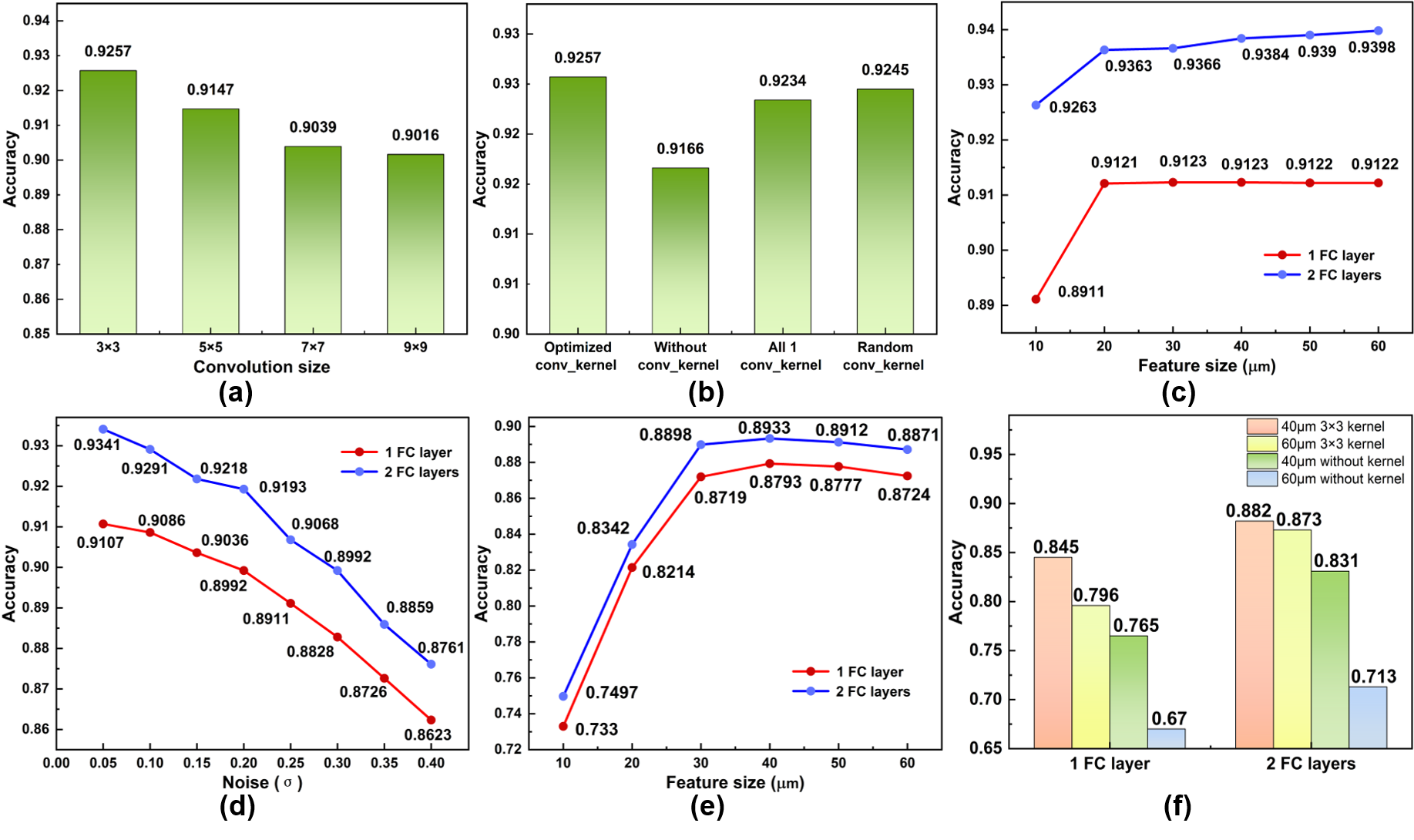


**Fig. S4 Simulation and validation results of single-kernel system. (a) Recognition accuracy map of different convolution kernel sizes. The network chosen was a 1 FC layer architecture. (b) Recognition accuracy map of different convolution kernels of size 3 × 3. The network chosen was a 1 FC layer architecture. (c) Curve of recognition accuracy variation with feature size (taking only diffraction into consideration). (d) Curve of recognition accuracy variation with Gaussian noise. The independent variable is Gaussian noise standard deviation. (e) Curve of recognition accuracy varies with feature size (taking diffraction, noise, and luminous flux into consideration). The standard deviation of the Gaussian noise was set to 0.2. (f) Recognition accuracy of different feature sizes in the experiment (1/10 of the dataset was used).**

Feature size is an essential factor that influences the classification accuracy of a task. Different feature sizes change the corresponding accuracy of the task. The feature size has a low threshold; however, it does not mean that the larger the feature size, the better the task performance. First, the entire luminous flux through the mask is proportional to the opening area of the mask, i.e., the square of the feature size. In addition, according to the principle of optical convolution, the size of the object is related to the system setup. When the distance relationship is fixed, the size of the object is proportional to the feature size. As shown in Fig. S1(b), the size of the object affects the contrast of the image captured by the sensor. The greater the object size, the more significant the difference between the light intensity values at the center and the edge of the image on the sensor, while the details at the edge may be ignored, thereby decreasing the accuracy. As shown in Fig. S4(e), when all three factors are considered, as the feature size increases, the recognition accuracy first increases and then decreases, and the highest point corresponds to a feature size of 40 μm. With the acquisition conditions, such as the exposure time, unchanged, we used 1/10 of the entire MNIST dataset for training and testing in the experiment. As shown in Fig. S3(f), the accuracy of the 40-μm $3\times3$ convolution kernel or no convolution kernel was higher than that of the 60-μm convolution kernel under the same conditions. Therefore, in the experiment with a single convolution kernel and multiple convolution kernels, we chose a feature size of 40 μm. Notably, the image displayed on the screen should be pre-compensated to maintain good contrast of the image captured by the sensor.

**Supplementary Note 6: Computational power and power consumption**

In the network for a specific task, operations such as convolution, pooling, BatchNorm, and ReLU are performed. These operations have corresponding computational power consumptions. Convolution consumes the highest proportion of power.

Floating-point operations (FLOPs) are recognized as amounts of calculation and can be used to measure the complexity of a model or algorithm, e.g., the number of floating-point operations of a neural network. If we take convolution as an example, the size of the object is $n\times n\times c_{1}$, and the size of the convolution kernel is $m\times m\times c_{2}$, where $c_{1}$and $c_{2}$ are the corresponding channel numbers. The FLOPs for one convolution operation consist of $m^{2}$ multiplications and $m^{2}-1$ additions. For channel numbers, there are $m^{2}\times c_{1}\times\left(n-m+1\right)^{2}\times c_{2}$ multiplications and $\left(m^{2}\times c_{1}-1\right)\times\left(n-m+1\right)^{2}\times c_{2}$ additions in the entire convolution layer. If bias is considered, the FLOPs for one convolution layer are $2\times m^{2}\times c_{1}\times\left(n-m+1\right)^{2}\times c_{2}$. Assuming that the thickness of the optical mask is $d$, the operational frequency $f$ and the number of operations per second (FLOPS) of our system are as follows:

$\begin{matrix} f=\frac{c}{d} \\ FLOPS=FLOPs\cdot f=\frac{2\times\left(m^{2}\times c_{1}-1\right)\times\left(n-m+1\right)^{2}\times c_{2}\times c}{d} \end{matrix}$ (S17)

where $c$is the speed of the light. It should be noted that if the size of the kernel is larger than the object, the expressions of $m$ and $n$ should be exchanged in Eq. (S17).

Based on the face recognition task, the pixel size of the image is $112\times112\times3$, the convolution kernel size is $510\times510$, and the thickness of the mask is; thus $d=2\text{ }mm$and the FLOPS of the optical convolution layer is $4.5\times10^{18}$, which is larger than that of many optical and electrical neural networks, as shown in Table S1.

**Table S1. Comparison of FLOPS.**

|  | Neural network | | | |
| --- | --- | --- | --- | --- |
|  | Electrical neural network | All-optical neural network | Hybrid opto-electrical neural network | Our work |
|  |  |  |  |  |
| FLOPS | 1.42×10^14^  (GeForce RTX 3080 Laptop) | 4×10^19^ [17] | 4×10^15^ [33]  6.2×10^14^ [30]  2.4×10^14^ [31] | 4.5×10^18^ |
|  |  | 3.76×10^16^ [19] |  |  |
|  |  | 8.4×10^15^ [18] |  |  |
|  |  | 3.2×10^12^ [22] |  |  |
|  |  | 1.19×10^10^ [29] |  |  |

**Supplementary Note 7: Camera imaging processing pipeline**

The raw measurements captured by the imaging sensor are degraded by many factors, such as noise, optical aberrations, sub-sampling on color-filter arrays, and cross-talk. To address these problems, conventional imaging uses a sensor to process raw data in a sequential pipeline of steps, such as denoising, demosaicing, color transformations, gamut mapping, tone mapping, and image compression. This process is called image signal processing (ISP). Existing imaging pipelines are designed for photography, which produce high-quality images for human vision, such as high-resolution, low-noise, and high-contrast images. Computer vision tasks such as classification and recognition, however, do not necessarily require the same high quality as humans. The raw data may be noisier than that after ISP, but it also provides more information. In addition, the total energy consumption from the image input to the classification output in our framework contains the consumption of the sensor itself, the ISP, and the suffix layers of the neural network. As the hardware acceleration reduces the cost of the neural network, the cost of capturing and processing images accounts for a larger share of the total system power consumption. Whether the ISP or other processes are necessary should be based on specific computer vision tasks. If the accuracy in dealing directly with the raw data is close to or the same as that dealing with the data after ISP, the framework performance can be obtained while the energy consumption can be further reduced, and the entire system will be more fully minimized.

**Supplementary Note 8: Noise model and noise estimation**

It is inevitable that there will be noise in the image capture process. When faced with a complex task such as human face recognition, a noise model is significant, which will help increase the network robustness and the task accuracy. In note 5, only Gaussian noise is discussed in the single-kernel and multiply-kernel systems. Here a more detailed noise model according to ref.1 is built to simulate the noise in the system.

**Noise model**

The raw data $D$ captured by a digital sensor can be expressed by a linear model

$D=KI+N$ (S18)

where $I$ is the number of photoelectrons that is proportional to the scene irradiation, $K$ represents the gain of the system which composes analog and digital gains, and $N$ denotes the whole noise caused by light and the sensor. A schematic diagram of the noise in the captured images process is shown in Fig.S5.

**
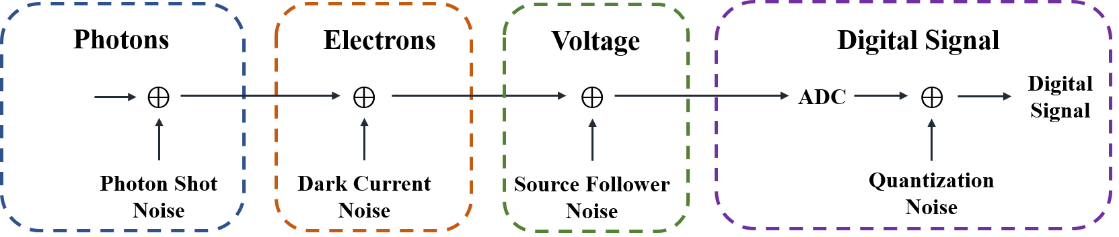
**

**Fig.S5. Schematic diagram of the noise in the captured images process.**

In our model, we consider the sensor imaging process to evaluate the noise. Three aspects should be considered to model noise: the conversions from the photons to electrons, from electrons to voltage, and from voltage to digital signal.

During exposure, incident light in the form of photons hits the sensor pixel area, which liberates photoelectrons proportional to the light intensity of the photoelectric effect. Due to the quantum nature of light, there exists an inevitable uncertainty in the number of electrons collected, which causes the ‘photon shot noise’. Such uncertainty follows a Poisson distribution, that is

$(I+N_{p})\sim P(I)$ (S19)

where $N_{p}$ is represented as the photon shot noise and $P$ denotes the Poisson distribution. It should be noted that photon shot noise depends on the light signal. It is a fundamental limitation and can not be avoided even for a perfect sensor.

There are other noise sources in the photon-to-electron process, such as the photo response uniformity and dark current noise. Due to the development of the CMOS sensor design and fabrication, the sensors now have a lower dark current and better photo response uniformity. According to ref.4, we put the dark current noise $N_{d}$ into the read noise $N_{read}$, and talk the whole read noise together.

After the transformed electrons are collected, they need to be integrated, amplified, and read out as measurable charge or voltage. The noise in this process includes thermal noise, source follower noise [2]. After researching the physical origin of these noises, we absorb the dark current noise $N_{d}$, thermal noise $N_{t}$, and source follower noise $N_{s}$ to describe the read noise:

$N_{read}=N_{d}+N_{t}+N_{s}$ (S20)

The read noise is generally assumed to follow a Gaussian distribution, and the mean is set to zero. Although zero-mean noise assumption is generally used in most noise evaluation situations, under extreme low-light settings, the system has the non-negligible direct current. This component originates from the dark current noise, making the noise distribution no longer zero-centered. So we model the DC noise component as the mean value of the read noise distribution. The read noise should follow:

$N_{read}\sim G(\mu,\sigma)$ (S21)

where $\mu$ denote the DC noise component, $G$represents the Gaussian distribution, the mean and the variation should be estimated on the dark field.

To generate an image, the analog voltage signal read out during the last stage is quantized into discrete codes using an analog-to-digital converter (ADC), which causes quantization noise. Quantization noise is a rounding error between the analog voltage and the final digital value, which can be assumed to follow a uniform distribution

$N_{q}\text{\textasciitilde}U(-1/2q,1/2q)$ (S22)

where $U$ denotes the uniform distribution over the range $[-1/2q,1/2q]$ and $q$is the quantization step.

To sum up, our noise model consists of three components:

$N=KN_{p}+N_{read}+N_{q}$ (S23)

**Noise** **estimation**

The estimation of the noise parameters is discussed as follows. Our calibration method uses two types of raw data, i.e., the dark field images and the uniformly illuminated images. It is worth noting that the estimated noise is under a fixed exposure time. The dark field images are captured under a dark environment without a light source, and the uniformly illuminated images are captured when the sensor is uniformly illuminated by a LED light source. The uniformly illuminated images characterize the light-dependent photon shot noise, thus can be used to estimate the system gain $K$, while the dark field images describe the noise independent of light, which can be used to derive the other parameters $\mu$, $\sigma$.

1. Estimate $K$ for photon shot noise.

According to the equation (S18)(S19)(S23) above, the raw data captured by the sensor can be expressed by

$D=K(I+N_{p})+N_{read}+N_{q}$ (S24)

So the variance of the noisy raw data is given by

$Var(D)=K^{2}Var(I+N_{p})+Var(N_{read}+N_{q})$ (S25)

where $Var(\cdot)$ denotes the variance operator that calculates the variance of a random variable. As discussed above, $(I+N_{p})$ follows a Poisson distribution, whose variation equals to its mean, so we can get

$Var(D)=K^{2}I+Var(N_{read}+N_{q})=K(KI)+Var(N_{read}+N_{q})$ (S26)

where is the actual signal represented by digital numbers.

It can be concluded that there is a linear relationship between the signal variance $Var(D)$ and the underlying clean digital signal $KI$. When fitting the line of the variance $Var(D)$ and digital signal $KI$, the system gain $K$ can be determined. In the experiment, the actual signal value $KI$ can be approximated by the mean value of the captured raw images owing to the uniformity.

2. Estimate $\mu$ and $\sigma$ for read noise.

Given the dark field images, the DC noise component can be calculated by averaging all pixel values and frames captured at different times; the average value can be regarded as $\mu$. And the variance $\sigma$ can be obtained based on the same method. The average $\mu$ and variance $\sigma$ are added as a Gaussian distribution to simulate the read noise.

Now some examples in the experiment is shown to demonstrate the noise model. The exposure time is set to 5 ms. We captured the dark field and uniformly illuminated images at three gain settings, and the ADC process is at 16-bit depth. The goodness of fit $R^{2}$ is used to evaluate the accuracy of the simulated noise. A higher $R^{2}$ indicates a better fit.

The estimate of the gain is shown in Fig.S6. As we can see, these data points can be better fitted to a straight line, whose slope characterizes the overall system gain $K$ at the fixed exposure time setting of the sensor.

**
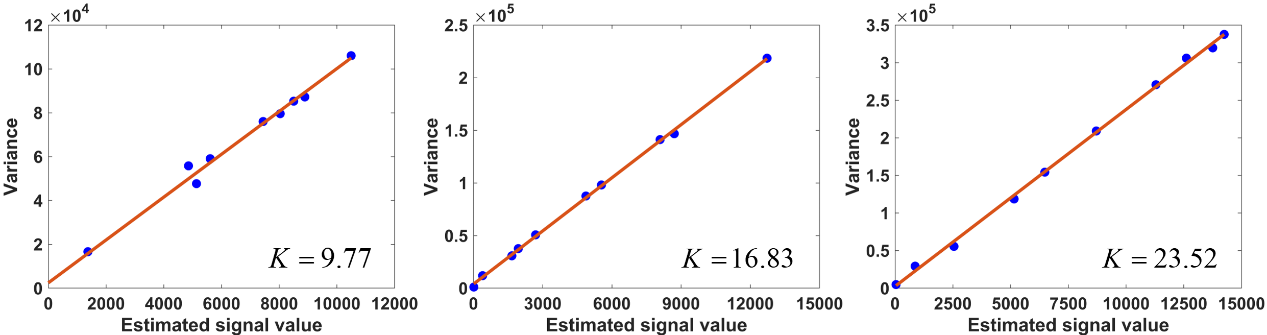
**

**Fig.S6.** **Estimation of the overall system gain $K$ under three different gain settings. The signal variance and the signal value satisfy a linear function.**

After the estimation of system gain $K$ from the uniformly illuminated images and the parameters of the read noise from the dark field images, the parameters are used to simulate the noise. A raw image captured is used as the raw data; we can simulate photon shot noise by firstly converting a raw digital into the numbers of photoelectrons, then impose the corresponding Poisson, Gaussian, uniformly distribution noise on it, and finally revert it to the raw data and compared with the image captured by the sensor.

The visual comparisons of real and simulated noise images are presented in Fig.S7. Our model is capable of synthesizing relatively realistic noise at different system gains, which outperforms only added Gaussian noise model both in terms of the goodness-of-fit measure (i.e., $R^{2}$) and the visual similarity to real noise.

As we can see in Fig.S7, the Gaussian noise has been able to simulate noise relatively well in many situations, so we only put the Gaussian noise in the single and multiply kernels system to demonstrate the effect of noise on the network optimization and task recognition accuracy (shown in Fig.S4(d)). However, for a more complex vision task, a more accurate noise model is needed. So, our noise model is used in the large-kernel system for privacy-protecting face recognition task. The simulated noise is added into the optical convolution part (in Fig.S7) and the network reverse optimization process, so the optimized optical mask considers the real environmental noise, while the network is more robust to the noise and the recognition accuracy is higher.

**
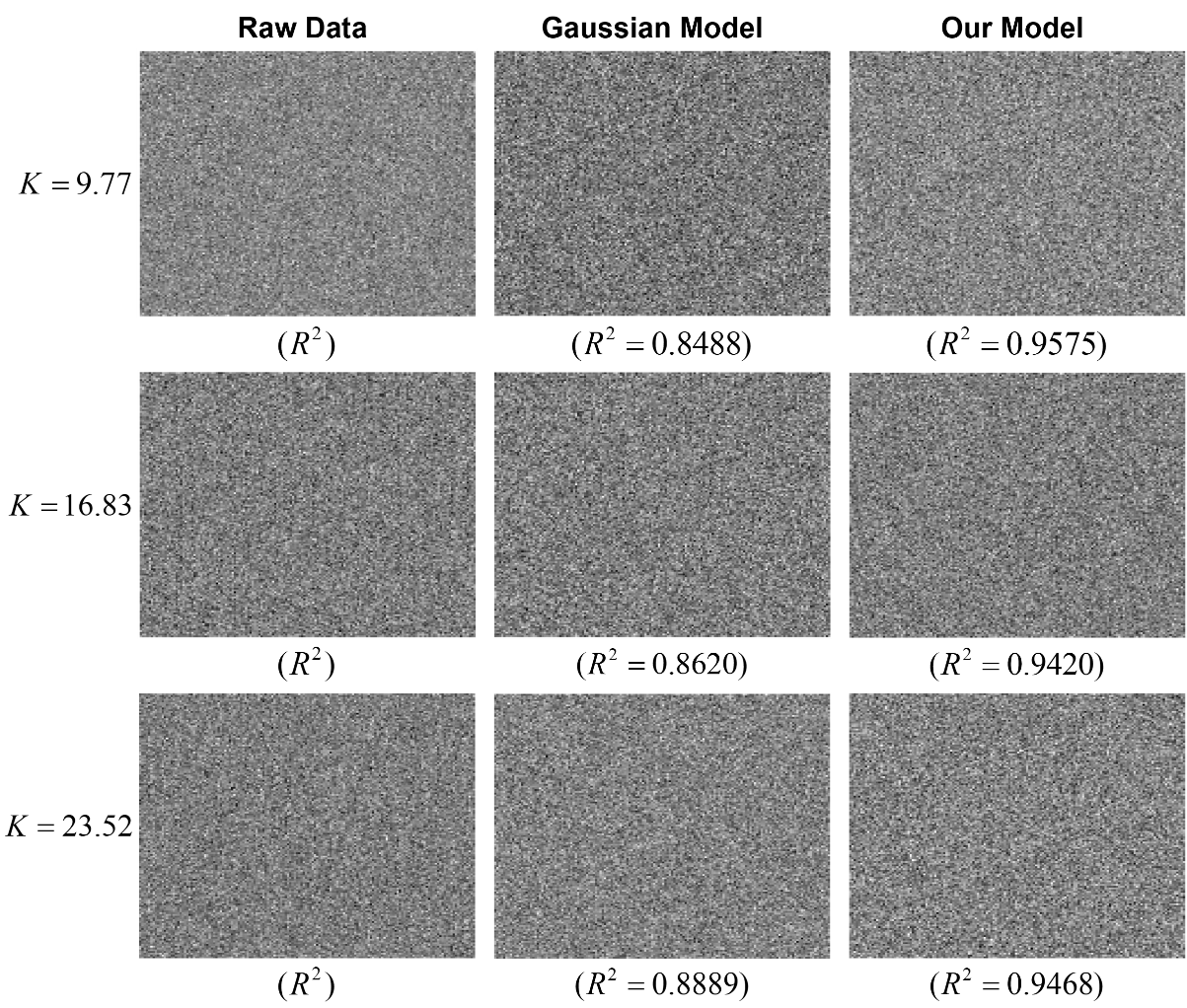
**

**Fig.S7. The comparison of the actual raw images and the simulated noise images based on Gaussian and our model. A higher $R^{2}$ indicates a better fit of the actual noise.**

**Supplementary Note 9: Potentials for 3D objects recognition/processing**

In the manuscript, the designed mask is only for a specific depth after calibration. As shown in Fig.S3(b), according to the triangle similarity theory, the following relationship can be obtained:

$\left\{\begin{matrix} S_{1}\text{=}M\cdot\frac{d_{1}+d_{MS}}{d_{1}} \\ S_{2}\text{=}M\cdot\frac{d_{2}+d_{MS}}{d_{2}} \\ \frac{S_{2}}{S_{1}}=\frac{d_{1}}{d_{2}}\cdot\frac{d_{2}+d_{MS}}{d_{1}+d_{MS}} \end{matrix}\right.$ (S27)

$\frac{S_{2}}{S_{1}}$represents the change proportion of PSF when the depth is changed. Take the face recognition task as an example, the distance between the human face and the mask is about 164 mm, and the depth of human face is about 5cm. When $d_{1}$ is chosen as 164 mm and $d_{2}$ is chosen as 214mm, $d_{MS}$ for the sensor is 1.02 mm. After calculation, we can obtain the proportion $\frac{S_{2}}{S_{1}}$is 0.9986. The conclusion can be proved that the kernel under different depths is almost the same within the face depth. While the network has robustness, the depth of the human face basically does not affect the final recognition results.

In the large-kernel experiment, a picture of 6.6cm*6.4cm is displayed on the screen. We set the position at a distance of 15.5cm from the mask as the center calibration plane, then translate the picture 5cm forward and backward along with the center calibration plane. The capture taken at different locations and the PSF of the center calibration plane are utilized to restore the image. Assuming a calibrated PSF can be obtained at the center calibration plane and $H$ is the Fourier transform of this PSF, in our experiments, we initialize $W$ using $F^{-1}(\frac{H^{*}}{K+\left|H\right|^{2}})$, which is the well-known Wiener filter. In this expression, $K$ is a regularization parameter related to the signal-to-noise ratio. Meanwhile, the PSF on the matching calibration plane is applied and compare the two restored results.


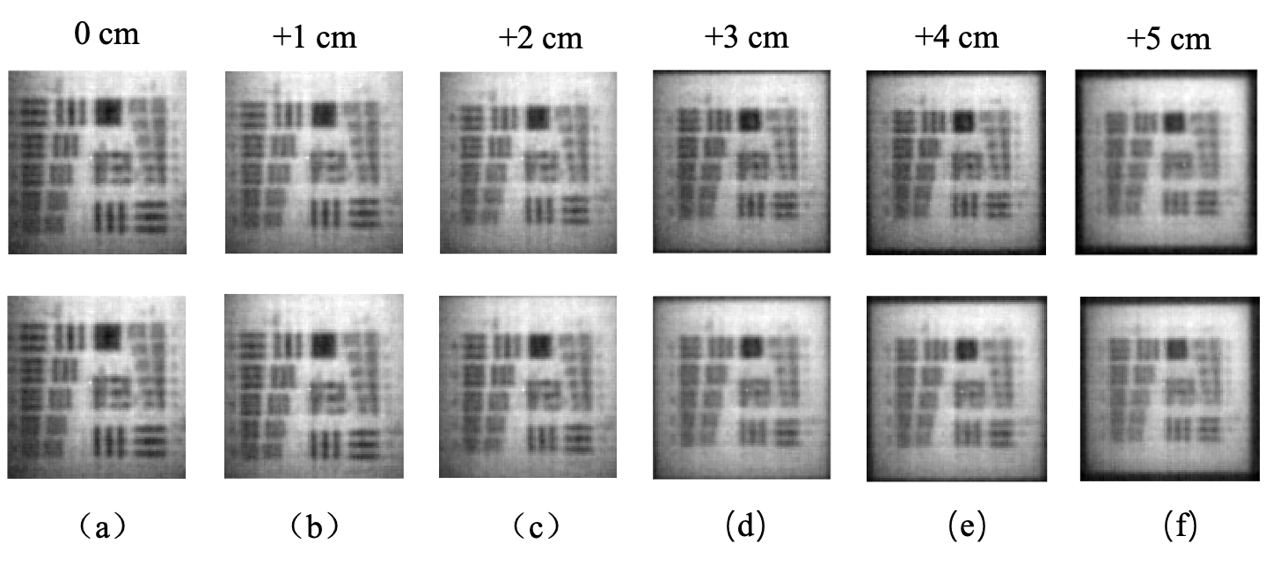


**Fig.S8. During moving backward 5 cm from the center position, comparing with the images restored by each position applying the PSF of this position or the center calibration plane. The first row was captured with an accurate PSF that matches the position. The second row was captured with the PSF of the center calibration plane. (a) captured at the center calibration plane. (b) captured at 1cm behind the center calibration plane. (c) captured at 2 cm behind the center calibration plane. (d) captured at 3 cm behind the center calibration plane. (e) captured at 4 cm behind the center calibration plane. (f) captured at 5 cm behind the center calibration plane.**


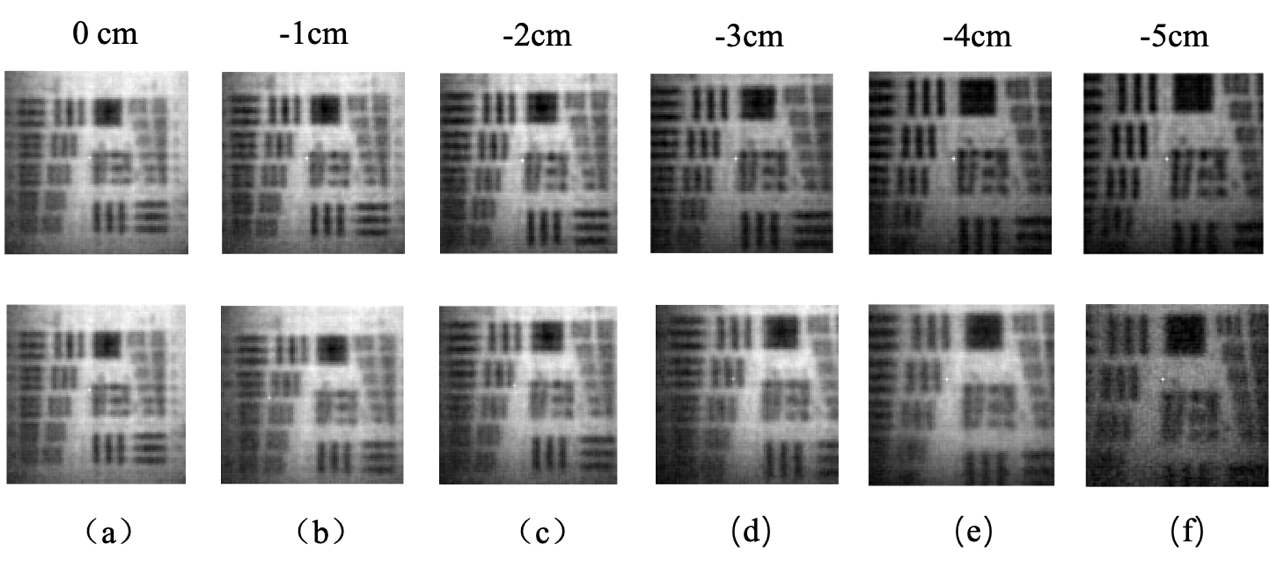


**Fig.S9. During moving forward 5 cm from the center position, comparing with the images restored by each position applying the PSF of this position or the center calibration plane. The first row was captured with an accurate PSF that matches the position. The second row was captured with the PSF of the center calibration plane. (a) captured at the center calibration plane. (b) captured at 1 cm in front of the center calibration plane. (c) captured at 2 cm in front of the center calibration plane. (d) captured at 3 cm in front of the center calibration plane. (e) captured at 4 cm in front of the center calibration plane. (f) captured at 5 cm in front of the center calibration plane.**

From the above results shown in Fig.S8 and Fig.S9, it can be analyzed that the restored pictures taken at different distances have the effect of scaling. However, at the same position, whether to use the calibration PSF of this position has little effect, the network has the ability to accommodate this error, and a specific range of depth of field also increases the robustness of the network.

When imaging 3D Objects with Lensless Systems, there have already been many researchers set up 3D PSFs to reconstruct the 3D objects. For example, Antipa et. al used a diffuser to be the encoding devices. The speckle patterns at different depths are collected and combined to be the 3D PSFs [3]. And the 3D objects (such as biological samples) can be reconstructed. In the manuscript, we complete the vision task by photographing 3D objects. In vision tasks, such as recognizing 3D objects, we can collect the PSFs (large convolution kernels) at different depths as prior and send them into the network training process. Before testing, some actual 3D objects should be captured to fine-tune the network, and the network can reconstruct 3D information of the objects.

**Supplementary Note 10: Potentials of processing RGB images**

In the manuscript, we chose the gray value image as the object because the mask is also gray value. If the objects are RGB images, the optical convolution calculation can also be made in current architecture. However, the convolution kernels for the different color channels (RGB) are the same, so the convolution results for the RGB images or the gray value images are the same.

In the future, we can design the mask for RGB images, in which various color channels correspond to different kernels. The schematic diagram of the mask for RGB images is as follows. As shown in Fig.S10, the convolution kernels for the different color channels are independent. When the kernels for R, G, and B channels are spatially aliased, the mask can deal with RGB images. For example, the mask can be fabricated by filter arrays with varying transmission spectra. While in this situation, we can deal with the RGB images using the grayscale sensor, which will decrease the manufacturing product and difficulty of the sensor.

**
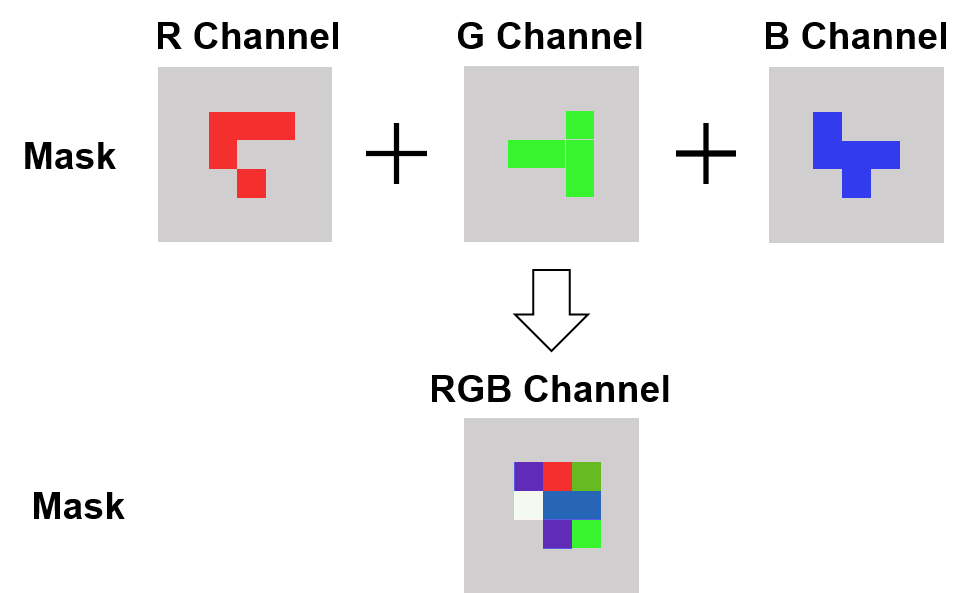
**

**Fig.S10. The schematic diagram of the mask for RGB images.**

**Supplementary Note 11: Neural network training and experiment results in the** **Face Recognition task**

During training, the measured PSF at the calibration plane is used as the initial value for the trainable inversion. U-Net is trained of Adam optimizer with a learning rate of 0.0001, a batch size of 4, and 5 epochs. Inception-ResNet-v1 is trained of Adam optimizer and 100 epochs. We use the model pre-trained on ImageNet as the initial weights. In the first 50 epochs, we freeze backbone network parameters. It is trained with an initial learning rate of 0.001, a batch size of 64. In the last 50 epochs, we update backbone network parameters. It is trained with an initial learning rate of 0.0001, a batch size of 32. The Loss curve is shown in Fig.S11.

**
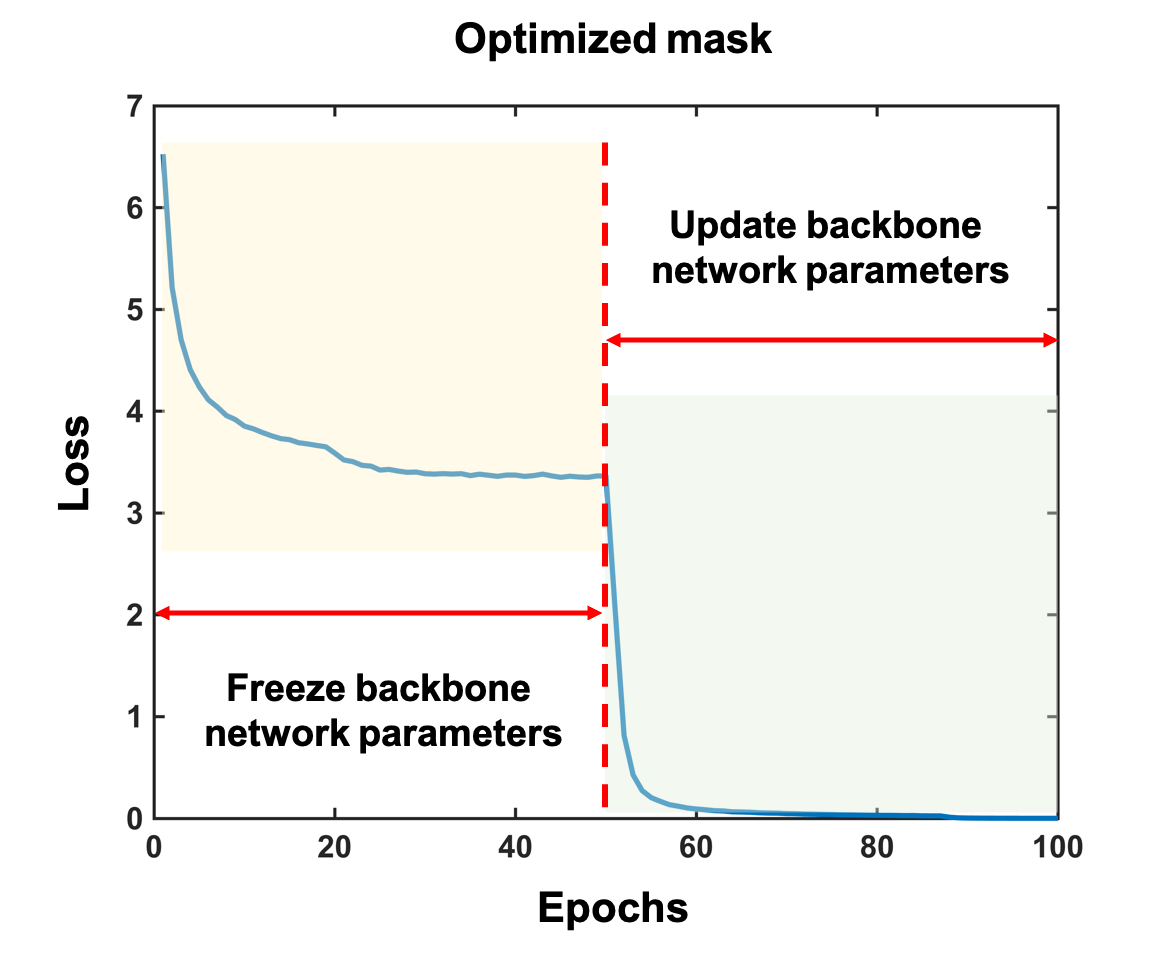
**

**Fig.S11. Loss curve during backbone network (Inception-ResNet-v1) training with the optimized mask in the experiment. We freeze backbone network parameters in the first 50 epochs, then update backbone network parameters in the last 50 epochs.**

As shown in Table S2, by comparing accuracy, precision, recall and F1-score, the optimized pattern performs better than the MLS pattern in the privacy-preserving face recognition task.

Table S2. Experiment results in the Face Recognition task.

| Method | Accuracy | Precision  (Weighted average) | Recall  (Weighted average) | F1-score  (Weighted average) |
| --- | --- | --- | --- | --- |
| MLS pattern | 65.1% | 69% | 66% | 0.64 |
| Optimized pattern | 71.6% | 75% | 72% | 0.70 |

**References**

1. Wei, K., Fu, Y., Zheng, Y. & Yang, J. Physics-based Noise Modeling for Extreme Low-light Photography. IEEE Transactions on Pattern Analysis and Machine Intelligence (2021).

2. Gow, R. D. et al. A comprehensive tool for modeling CMOS image-sensor-noise performance. Ieee T Electron Dev 54, 1321-1329 (2007)

3. Antipa, N. et al. DiffuserCam: lensless single-exposure 3D imaging. Optica 5, 1-9 (2018).
